# Supplementary material for: Longitudinal piezoelectric resonant photoelastic modulator for efficient intensity modulation at megahertz frequencies
Source: Nat Commun. 2022 Mar 22;13:1526. doi: 10.1038/s41467-022-29204-9 (PMC8941116; doi:10.1038/s41467-022-29204-9)
Supplement: Supplementary file 1 — Supplementary Information [file 41467_2022_29204_MOESM1_ESM.pdf]

Supplementary Information: Longitudinal piezoelectric resonant photoelastic modulator for efficient intensity modulation at megahertz frequencies

Okan Atalar[1*], Raphaël Van Laer[2,3], Amir H. Safavi-Naeini[2], and Amin Arbabian[1]

[1] *Department of Electrical Engineering, Stanford University, Stanford, California 94305, USA*
[2] *Department of Applied Physics and Ginzton Laboratory, Stanford University, Stanford, California 94305, USA*
[3] *Currently at Department of Microtechnology and Nanoscience (MC2), Chalmers University of Technology, Sweden*
[*] *Corresponding author: okan@stanford.edu*

Contents

1 Strain Profile in the Wafer **1**

2 Intensity Modulation of a Laser Beam **3**

3 Contribution of Electro-Optic Effect **10**

4 Intensity Modulation Efficiency **11**

5 s_{11} Measurement at Different RF Power Levels **12**

6 Intensity Modulation Efficiency of Different Modulation Mechanisms **13**

7 Strain Profile of Other Acoustic Modes **16**

8 Tolerance of Acoustic Mode to Variations **16**

9 Modulator Material Choice **16**

10 Reaching Higher Intensity Modulation Frequencies **17**

11 Ranging Performance of the Time-of-Flight Imaging System **17**
11.1 Ranging accuracy of the time-of-flight imaging system used for the experiments 19
11.2 Achievable ranging accuracy with single frequency operation 19
11.3 Achievable ranging accuracy with multi-frequency operation 19

1 Strain Profile in the Wafer

In this section, we show the strain profiles in the wafer when excited at the acoustic resonance frequency f_c through the surface electrodes. We then derive the modified index ellipsoid due to strain. When the wafer is excited at $f_c = 3.7696$ MHz with 2Vpp applied to the wafer electrodes, six different strain profiles are excited: S_{xx}, S_{yy}, S_{zz}, S_{xy}, S_{xz}, S_{yz}. These profiles are shown for three different planes at $y = 0.1$ mm, $y = 0.3$ mm, and $y = 0.45$ mm in Fig. S1 and Fig. S2, respectively.

To calculate the overall rotation of polarization, the volume average strain for a localized region is used. This is because the wafer can be thought of as consisting of thin sheets of infinitesimal thickness parallel to the wafer surface. The volume average strain captures the overall rotation of polarization after a plane wave passes through these infinitesimal regions. The index ellipsoid for lithium niobate (LN) can be expressed as[1]:

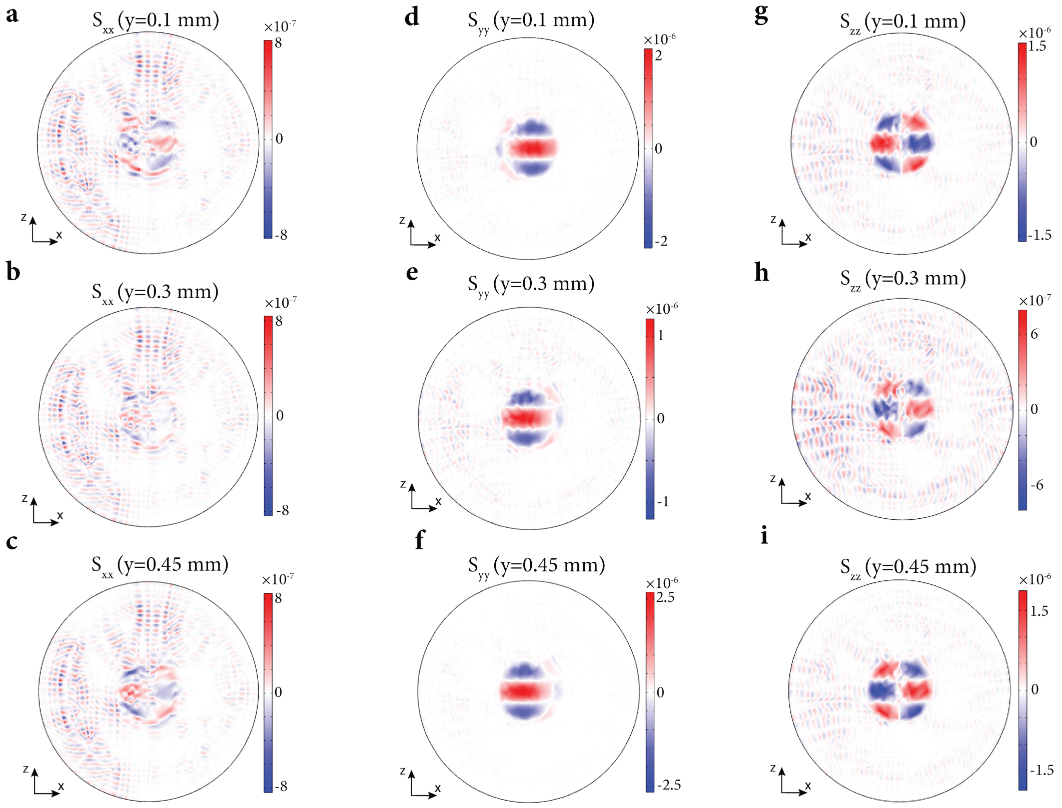

Fig. S1. Normal strain profiles in the wafer. **a** S_{xx} strain profile for the plane that is parallel and 0.1 mm above the bottom wafer surface. 2Vpp is applied to wafer surface electrodes. **b** S_{xx} strain profile for the plane that is parallel and 0.3 mm above the bottom wafer surface. 2Vpp is applied to wafer surface electrodes. **c** S_{xx} strain profile for the plane that is parallel and 0.45 mm above the bottom wafer surface. 2Vpp is applied to wafer surface electrodes. **d** S_{yy} strain profile for the plane that is parallel and 0.1 mm above the bottom wafer surface. 2Vpp is applied to wafer surface electrodes. **e** S_{yy} strain profile for the plane that is parallel and 0.3 mm above the bottom wafer surface. 2Vpp is applied to wafer surface electrodes. **f** S_{yy} strain profile for the plane that is parallel and 0.45 mm above the bottom wafer surface. 2Vpp is applied to wafer surface electrodes. **g** S_{zz} strain profile for the plane that is parallel and 0.1 mm above the bottom wafer surface. 2Vpp is applied to wafer surface electrodes. **h** S_{zz} strain profile for the plane that is parallel and 0.3 mm above the bottom wafer surface. 2Vpp is applied to wafer surface electrodes. **i** S_{zz} strain profile for the plane that is parallel and 0.45 mm above the bottom wafer surface. 2Vpp is applied to wafer surface electrodes.

$$
\begin{aligned}
x^2\Big(\frac{1}{n_o^2}+p_{11}\bar{S}_{xx}+p_{12}\bar{S}_{yy}+p_{13}\bar{S}_{zz}+2p_{14}\bar{S}_{yz}\Big)+y^2\Big(\frac{1}{n_o^2}+p_{12}\bar{S}_{xx}+p_{11}\bar{S}_{yy}+p_{13}\bar{S}_{zz}-2p_{14}\bar{S}_{yz}\Big)+\\
z^2\Big(\frac{1}{n_e^2}+p_{31}\bar{S}_{xx}+p_{31}\bar{S}_{yy}+p_{33}\bar{S}_{zz}\Big)+2yz\Big(p_{41}\bar{S}_{xx}-p_{41}\bar{S}_{yy}+2p_{44}\bar{S}_{yz}\Big)+\\
2zx\Big(2p_{44}\bar{S}_{xz}+2p_{41}\bar{S}_{xy}\Big)+2xy\Big(2p_{14}\bar{S}_{xz}+(p_{11}-p_{12})\bar{S}_{xy}\Big)=1 \qquad \text{(S1)}
\end{aligned}
$$

To simplify the derivations, only the dominant strain distribution S_{yz} contributing the most to the photoelastic interaction is used. Using the photoelastic coefficients from[2], this approximation simplifies the expression to:

$$
x^2\Big(\frac{1}{n_o^2}+2p_{14}\bar{S}_{yz}\Big)+y^2\Big(\frac{1}{n_o^2}-2p_{14}\bar{S}_{yz}\Big)+z^2\Big(\frac{1}{n_e^2}\Big)+2yz\Big(2p_{44}\bar{S}_{yz}\Big)=1 \qquad \text{(S2)}
$$

We apply a rotation to the yz axis such that the new form is diagonal[1]. Using the coordinate transformations in equation (S3), equation (S2) can be transformed into equation (S4).

$$
\begin{aligned}
y &= y'\cos\theta - z'\sin\theta\\
z &= y'\sin\theta + z'\cos\theta \qquad \text{(S3)}
\end{aligned}
$$

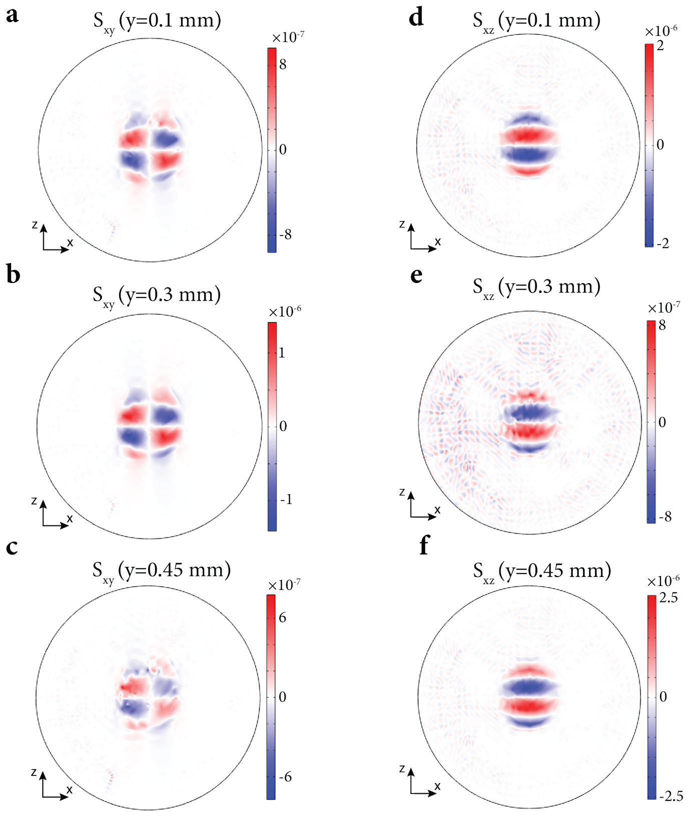

Fig. S2. Shear strain profiles in the wafer. **a** S_{xy} strain profile for the plane that is parallel and 0.1 mm above the bottom wafer surface. 2Vpp is applied to wafer surface electrodes. **b** S_{xy} strain profile for the plane that is parallel and 0.3 mm above the bottom wafer surface. 2Vpp is applied to wafer surface electrodes. **c** S_{xy} strain profile for the plane that is parallel and 0.45 mm above the bottom wafer surface. 2Vpp is applied to wafer surface electrodes. **d** S_{xz} strain profile for the plane that is parallel and 0.1 mm above the bottom wafer surface. 2Vpp is applied to wafer surface electrodes. **e** S_{xz} strain profile for the plane that is parallel and 0.3 mm above the bottom wafer surface. 2Vpp is applied to wafer surface electrodes. **f** S_{xz} strain profile for the plane that is parallel and 0.45 mm above the bottom wafer surface. 2Vpp is applied to wafer surface electrodes. **g** S_{yz} strain profile for the plane that is parallel and 0.1 mm above the bottom wafer surface. 2Vpp is applied to wafer surface electrodes. **h** S_{yz} strain profile for the plane that is parallel and 0.3 mm above the bottom wafer surface. 2Vpp is applied to wafer surface electrodes. **i** S_{yz} strain profile for the plane that is parallel and 0.45 mm above the bottom wafer surface. 2Vpp is applied to wafer surface electrodes.

$$x^2\left(\frac{1}{n_o^2}+2p_{14}\bar{S}_{yz}\right)+y'^2\left(\frac{1}{n_o^2}-2p_{14}\bar{S}_{yz}+(2p_{44}\bar{S}_{yz})\tan\theta\right)+z'^2\left(\frac{1}{n_e^2}-(2p_{44}\bar{S}_{yz})\tan\theta\right)=1$$

$$\tan(2\theta)=\frac{4p_{44}\bar{S}_{yz}}{\left(\frac{1}{n_o^2}-2p_{14}\bar{S}_{yz}\right)-\left(\frac{1}{n_e^2}\right)} \tag{S4}$$

Since $\tan\theta \ll 1$ in most cases ($\bar{S}_{yz} < 10^{-3}$ in experiments), we neglect the modulations of the y' and z' axis which include the $\tan\theta$ term. The wafer can effectively be treated as three separate regions. The blue regions in Fig. S2**g,h,i** are anti-phase with the red region.

$$x^2\left(\frac{1}{n_o^2}+2p_{14}\bar{S}_{yz}\right)+y'^2\left(\frac{1}{n_o^2}-2p_{14}\bar{S}_{yz}\right)+z'^2\left(\frac{1}{n_e^2}\right)=1 \tag{S5}$$

The wafer therefore functions as a polarization modulator at the applied frequency f_c. To simplify the derivations (and since $\tan\theta << 1$), we assume $y' \approx y$ and $z' \approx z$ for the remainder of this document.

2 Intensity Modulation of a Laser Beam

To convert the polarization modulator into an intensity modulator, the wafer is placed between polarizers. To find the response of an arbitrary laser beam passing through the polarizer, wafer, and polarizer, it is suffi-

cient to find the intensity modulation profile for each plane wave (k-space), since a laser beam can be written as the linear superposition of plane waves. We will treat the propagation of a plane wave with wave vector $\hat{k} = \hat{a}_x \sin\theta\cos\psi + \hat{a}_y \sin\theta\sin\psi + \hat{a}_z \cos\theta$, with random polarization, and intensity I_0 through the three elements separately. In each step, we will keep track of the polarization and intensity of the plane wave. The two polarizers have transmission axis $\hat{t} = \frac{\hat{a}_x + \hat{a}_z}{\sqrt{2}}$. The totally blocked polarization by the first polarizer is given by:

$$\bar{p}_1 = \hat{t} \times \hat{k} = \hat{a}_x(-\sin\theta\sin\psi) + \hat{a}_y(\sin\theta\cos\psi - \cos\theta) + \hat{a}_z(\sin\theta\sin\psi) \tag{S6}$$

The transmitted polarization is found as $\bar{p}_2 = \bar{p}_1 \times \hat{k}$.

$$\bar{p}_2 = \hat{a}_x(\sin\theta\cos\theta\cos\psi - \cos^2\theta - \sin^2\theta\sin^2\psi) + \hat{a}_y(\sin^2\theta\cos\psi\sin\psi + \sin\theta\cos\theta\sin\psi) + \hat{a}_z(\sin\theta\cos\theta\cos\psi - \sin^2\theta) \tag{S7}$$

Assuming random polarization for the incoming plane wave, the plane wave has intensity $\frac{I_0}{2}$ and polarization $\bar{p}_2$ after propagating through the first polarizer. Now we turn our attention to the propagation of the plane wave through the wafer. To simplify the calculations, we will first solve for the static case (no voltage applied to the wafer electrodes, and therefore no strain in the wafer).

When a plane wave has oblique incidence on a uniaxial planar medium such as lithium niobate, an ordinary and an extraordinary wave is excited. To find the refraction angles and the effective refractive indices for these two possible solutions, we will use phase-matching. Let ϕ denote the angle between the surface normal of the wafer ($\hat{a}_y$) and $\hat{k}$. We can therefore write $\cos\phi = \hat{k} \cdot \hat{a}_y = \sin\theta\sin\psi$, from which we find: $\phi = \cos^{-1}(\sin\theta\sin\psi)$. Applying phase-matching for the ordinary and extraordinary waves:

$$\sin\phi = n_o \sin\tilde{\theta}_o = n_e(\theta_e)\sin\tilde{\theta}_e \tag{S8}$$

In equation (S8), $\tilde{\theta}_o$ is the angle between $\hat{a}_y$ and the refracted ordinary wave vector ($\hat{k}_{ro}$), θ_e is the angle between $\hat{a}_z$ and the refracted extraordinary wave vector ($\hat{k}_{re}$), $\tilde{\theta}_e$ is the angle between $\hat{a}_y$ and $\hat{k}_{re}$. The extraordinary refractive index is expressed as: $\frac{1}{n_e^2(\theta_e)} = \frac{\cos^2\theta_e}{n_o^2} + \frac{\sin^2\theta_e}{n_e^2}$.

The plane of incidence for the refraction problem is: $\bar{v} = \hat{a}_y \times \hat{k} = -\hat{a}_z \sin\theta\cos\psi + \hat{a}_x \cos\theta$. We know that the refracted waves will lie on the plane of incidence. Therefore, $\hat{k}_{re} \cdot \bar{v} = \hat{k}_{ro} \cdot \bar{v} = 0$, and by definition of the angles, the following expressions are true: $\hat{k}_{re} \cdot \hat{a}_z = \cos\theta_e$ and $\hat{k}_{re} \cdot \hat{a}_y = \cos\tilde{\theta}_e$. We first solve for the extraordinary wave vector $\hat{k}_{re} = a_1\hat{a}_x + a_2\hat{a}_y + a_3\hat{a}_z$. We use the following expressions to find $\hat{k}_{re}$:

$$\begin{gathered} \hat{k}_{re} \cdot \bar{v} = a_1\cos\theta - a_3\sin\theta\cos\psi = 0 \\ \hat{k}_{re} \cdot \hat{a}_z = a_3 = \cos\theta_e \\ \hat{k}_{re} \cdot \hat{a}_y = a_2 = \cos\tilde{\theta}_e \\ |\hat{k}_{re}| = \sqrt{a_1^2 + a_2^2 + a_3^2} = 1 \end{gathered} \tag{S9}$$

We can use the above expressions to find:

$$\begin{gathered} a_1 = a_3\tan\theta\cos\psi = \cos\theta_e\tan\theta\cos\psi \\ \cos^2\theta_e\tan^2\theta\cos^2\psi + \cos^2\tilde{\theta}_e + \cos^2\theta_e = 1 \end{gathered} \tag{S10}$$

Using the expression above, we can arrive at: $\sin\tilde{\theta}_e = \cos\theta_e\sqrt{\tan^2\theta\cos^2\psi + 1}$. Inserting this expression into the extraordinary phase-matching condition: $\sin\phi = \frac{\cos\theta_e\sqrt{\tan^2\theta\cos^2\psi+1}}{\sqrt{\frac{\cos^2\theta_e}{n_o^2} + \frac{\sin^2\theta_e}{n_e^2}}}$.

To solve this, let $x = \cos\theta_e$. The phase-matching condition can now be expressed as: $\frac{\sin\phi}{\sqrt{\tan^2\theta\cos^2\psi+1}} = \frac{x}{\sqrt{\frac{x^2}{n_o^2} + \frac{1-x^2}{n_e^2}}}$. We therefore find the two angles as:

$$\theta_e = \cos^{-1}\left(\frac{n_o\sin\phi}{\sqrt{n_o^2 n_e^2(\tan^2\theta\cos^2\psi + 1) - n_e^2\sin^2\phi + n_o^2\sin^2\phi}}\right) \tag{S11}$$

$$\tilde{\theta}_e = \sin^{-1}\left(\frac{n_o\sin\phi\sqrt{\tan^2\theta\cos^2\psi + 1}}{\sqrt{n_o^2 n_e^2(\tan^2\theta\cos^2\psi + 1) - n_e^2\sin^2\phi + n_o^2\sin^2\phi}}\right) \tag{S12}$$

We now solve for the ordinary wave vector $\hat{k}_{ro} = b_1\hat{a}_x + b_2\hat{a}_y + b_3\hat{a}_z$. We apply the same strategy we used to solve the extraordinary wave case to solve for the ordinary wave, where θ_o is the angle between $\hat{a}_z$ and the refracted ordinary wave vector ($\hat{k}_{ro}$). We use the following to find $\hat{k}_{ro}$:

$$\begin{aligned}\hat{k}_{ro}\cdot\bar{v} &= b_1\cos\theta - b_3\sin\theta\cos\psi = 0\\ \hat{k}_{ro}\cdot\hat{a}_z &= \cos\theta_o\\ \hat{k}_{ro}\cdot\hat{a}_y &= \cos\tilde{\theta}_o\\ |\hat{k}_{ro}| &= \sqrt{b_1^2+b_2^2+b_3^2} = 1\end{aligned}\tag{S13}$$

Using the expressions above, we can write:

$$\begin{aligned}b_1 = b_3\tan\theta\cos\psi &= \cos\theta_o\tan\theta\cos\psi\\ \cos^2\theta_o\tan^2\theta\cos^2\psi + \cos^2\tilde{\theta}_o &+ \cos^2\theta_o = 1\end{aligned}\tag{S14}$$

We can now arrive at $\sin\tilde{\theta}_o = \cos\theta_o\sqrt{\tan^2\theta\cos^2\psi+1}$. Inserting this expression into the ordinary phase-matching condition: $\sin\phi = n_o\cos\theta_o\sqrt{\tan^2\theta\cos^2\psi+1}$. We find the two angles as:

$$\theta_o = \cos^{-1}\left(\frac{\sin\phi}{n_o\sqrt{\tan^2\theta\cos^2\psi+1}}\right)\tag{S15}$$

$$\tilde{\theta}_o = \sin^{-1}\left(\frac{\sin\phi}{n_o}\right)\tag{S16}$$

We will now find the polarizations $\bar{p}_o$ and $\bar{p}_e$ corresponding to the ordinary and extraordinary refracted waves, respectively.

$$\bar{p}_o = \hat{k}_{ro}\times\hat{a}_z = \cos\left(\sin^{-1}\left(\frac{\sin\phi}{n_o}\right)\right)\hat{a}_x - \frac{\sin\phi\tan\theta\cos\psi}{n_o\sqrt{\tan^2\theta\cos^2\psi+1}}\hat{a}_y\tag{S17}$$

$$\bar{p}_e = (\hat{k}_{re}\times\hat{a}_z)\times\hat{k}_{re} = -\cos^2\theta_e\tan\theta\cos\psi\hat{a}_x - \cos\theta_e\cos\tilde{\theta}_e\hat{a}_y + \left(\cos^2\tilde{\theta}_e + \cos^2\theta_e\tan^2\theta\cos^2\psi\right)\hat{a}_z\tag{S18}$$

We will now focus on finding the polarizations $\bar{p}_{oi}$ and $\bar{p}_{ei}$ in air incident to the wafer surface that correspond to the ordinary and extraordinary polarizations in the wafer: $\bar{p}_o$ and $\bar{p}_e$. We assume for simplifying the derivations that there is no reflection at the lithium niobate-air interface (ideal anti-reflection coating assumption). This assumption is fairly accurate if the incidence angle is nearly perpendicular to the wafer surface. This assumption allows us to simplify the refraction problem; polarization $\bar{p}_{oi}$ gets mapped to $\bar{p}_o$ in the wafer and polarization $\bar{p}_{ei}$ gets mapped to $\bar{p}_e$ in the wafer. We will first solve for the ordinary wave. Using the assumption of no reflection at the air-wafer boundary, we use the following to find $\bar{p}_{oi}$:

$$\begin{aligned}\bar{p}_{oi}\cdot\hat{k} &= 0\\ \bar{p}_o\cdot\bar{v} = \bar{p}_{oi}\cdot\bar{v} &= \frac{\cos\theta\cos\left(\sin^{-1}\left(\frac{\sin\phi}{n_o}\right)\right)}{\sqrt{\cos^2\left(\sin^{-1}\left(\frac{\sin\phi}{n_o}\right)\right)+\frac{\sin^2\phi\tan^2\theta\cos^2\psi}{n_o^2(\tan^2\theta\cos^2\psi+1)}}}\end{aligned}\tag{S19}$$

Let $\hat{p}_{oi} = c_1\hat{a}_x + c_2\hat{a}_y + c_3\hat{a}_z$, where $c_1^2+c_2^2+c_3^2 = 1$. We can now arrive at:

$$\begin{aligned}c_1\sin\theta\cos\psi + c_2\sin\theta\sin\psi + c_3\cos\theta &= 0\\ c_1\cos\theta - c_3\sin\theta\cos\psi &= \frac{\cos\theta\cos\left(\sin^{-1}\left(\frac{\sin\phi}{n_o}\right)\right)}{\sqrt{\cos^2\left(\sin^{-1}\left(\frac{\sin\phi}{n_o}\right)\right)+\frac{\sin^2\phi\tan^2\theta\cos^2\psi}{n_o^2(\tan^2\theta\cos^2\psi+1)}}}\end{aligned}\tag{S20}$$

Using the expressions above, we find c_2:

$$c_2 = \frac{-c_3(\cos\theta + \sin\theta\tan\theta\cos^2\psi)}{\sin\theta\sin\psi} - \frac{\cot\psi\cos\Big(\sin^{-1}\big(\frac{\sin\phi}{n_o}\big)\Big)}{\sqrt{\cos^2\Big(\sin^{-1}\big(\frac{\sin\phi}{n_o}\big)\Big) + \frac{\sin^2\phi\tan^2\theta\cos^2\psi}{n_o^2(\tan^2\theta\cos^2\psi+1)}}} \tag{S21}$$

Using $c_1^2+c_2^2+c_3^2 = 1$, c_3 can be expressed as the solution to the following quadratic equation: $\tilde{a}_1 c_3^2+\tilde{a}_2 c_3+\tilde{a}_3 = 0$, where:

$$\tilde{a}_1 = \tan^2\theta\cos^2\psi + \frac{(\cos\theta + \sin\theta\tan\theta\cos^2\psi)^2}{\sin^2\theta\sin^2\psi} + 1$$

$$\tilde{a}_2 = \frac{2\cos\Big(\sin^{-1}\big(\frac{\sin\phi}{n_o}\big)\Big)}{\sqrt{\cos^2\Big(\sin^{-1}\big(\frac{\sin\phi}{n_o}\big)\Big) + \frac{\sin^2\phi\tan^2\theta\cos^2\psi}{n_o^2(\tan^2\theta\cos^2\psi+1)}}}\left(\tan\theta\cos\psi + \frac{\cot\psi(\cos\theta + \sin\theta\tan\theta\cos^2\psi)}{\sin\theta\sin\psi}\right)$$

$$\tilde{a}_3 = \frac{\cos^2\Big(\sin^{-1}\big(\frac{\sin\phi}{n_o}\big)\Big) + \cot^2\psi\cos^2\Big(\sin^{-1}\big(\frac{\sin\phi}{n_o}\big)\Big)}{\cos^2(\sin^{-1}(\frac{\sin\phi}{n_o})) + \frac{\sin^2\phi\tan^2\theta\cos^2\psi}{n_o^2(\tan^2\theta\cos^2\psi+1)}} - 1 \tag{S22}$$

The solution to this quadratic equation is: $c_3 = \frac{-\tilde{a}_2 \pm \sqrt{\tilde{a}_2^2 - 4\tilde{a}_1\tilde{a}_3}}{2\tilde{a}_1}$ Since the quadratic equation can have two real solutions, how do we pick the right one? The solution that we pick is the one that maximizes $\hat{p}_{oi} \cdot \bar{p}_o$. The reason why two solutions emerge is because of the way we have framed the question. Specifically, using the equality $\hat{p}_o \cdot \bar{v} = \hat{p}_{oi} \cdot \bar{v}$, there are two angles satisfying the dot product equality. What we were trying to enforce was that the angle between $\hat{p}_o$ and $\bar{v}$ is equal to the angle between $\hat{p}_{oi}$ and $\bar{v}$, since no reflection at the boundary was assumed. We can now find c_2 by plugging c_3 into equation (S21). c_1 is found as:

$$c_1 = \sqrt{1 - c_2^2 - c_3^2} \tag{S23}$$

We will now find $\hat{p}_{ei}$. Let $\hat{p}_{ei} = d_1\hat{a}_x + d_2\hat{a}_y + d_3\hat{a}_z$, where $d_1^2 + d_2^2 + d_3^2 = 1$ and $\bar{p}_{ei} = \hat{p}_{oi} \times \hat{k}$. We can find the components as follows:

$$d_1 = \frac{c_2\cos\theta - c_3\sin\theta\sin\psi}{\sqrt{(c_2\cos\theta - c_3\sin\theta\sin\psi)^2 + (-c_1\cos\theta + c_3\sin\theta\cos\psi)^2 + (c_1\sin\theta\sin\psi - c_2\sin\theta\cos\psi)^2}} \tag{S24}$$

$$d_2 = \frac{-c_1\cos\theta + c_3\sin\theta\cos\psi}{\sqrt{(c_2\cos\theta - c_3\sin\theta\sin\psi)^2 + (-c_1\cos\theta + c_3\sin\theta\cos\psi)^2 + (c_1\sin\theta\sin\psi - c_2\sin\theta\cos\psi)^2}} \tag{S25}$$

$$d_3 = \frac{c_1\sin\theta\sin\psi - c_2\sin\theta\cos\psi}{\sqrt{(c_2\cos\theta - c_3\sin\theta\sin\psi)^2 + (-c_1\cos\theta + c_3\sin\theta\cos\psi)^2 + (c_1\sin\theta\sin\psi - c_2\sin\theta\cos\psi)^2}} \tag{S26}$$

We can express $\hat{p}_2$ as a linear superposition of $\bar{p}_{oi}$ and $\bar{p}_{ei}$. Specifically, $\hat{p}_2 = c_o\hat{p}_{oi} + c_e\hat{p}_{ei}$, where c_o and c_e denote the ordinary and extraordinary wave amplitudes in air, respectively. We therefore find $c_o = \hat{p}_2 \cdot \hat{p}_{oi}$ and $c_e = \hat{p}_2 \cdot \hat{p}_{ei}$.

$$c_o = \frac{c_1(\sin\theta\cos\theta\cos\psi - \cos^2\theta - \sin^2\theta\sin^2\psi) + c_2(\sin^2\theta\cos\psi\sin\psi + \sin\theta\cos\theta\sin\psi) + c_3(\sin\theta\cos\theta\cos\psi - \sin^2\theta)}{\sqrt{(\sin\theta\cos\theta\cos\psi - \cos^2\theta - \sin^2\theta\sin^2\psi)^2 + (\sin^2\theta\cos\psi\sin\psi + \sin\theta\cos\theta\sin\psi)^2 + (\sin\theta\cos\theta\cos\psi - \sin^2\theta)^2}} \tag{S27}$$

$$c_e = \frac{d_1(\sin\theta\cos\theta\cos\psi - \cos^2\theta - \sin^2\theta\sin^2\psi) + d_2(\sin^2\theta\cos\psi\sin\psi + \sin\theta\cos\theta\sin\psi) + d_3(\sin\theta\cos\theta\cos\psi - \sin^2\theta)}{\sqrt{(\sin\theta\cos\theta\cos\psi - \cos^2\theta - \sin^2\theta\sin^2\psi)^2 + (\sin^2\theta\cos\psi\sin\psi + \sin\theta\cos\theta\sin\psi)^2 + (\sin\theta\cos\theta\cos\psi - \sin^2\theta)^2}} \tag{S28}$$

Now we will calculate the refractive indices experienced by the ordinary and extraordinary waves when the wafer is excited through its surface electrodes with frequency f_c, leading to volume average strain $\bar{S}_{yz}$ in the wafer. To simplify the expressions, we assume that the polarization directions $\bar{p}_o$ and $\bar{p}_e$ do not change when strain is

present in the wafer. We will only calculate the change in the refractive indices using this assumption (due to change in the index ellipsoid via photoelasticity) to simplify the derivation. This assumption does not change the results significantly, since the dominant contribution is from the refractive index change.

We will first solve for the ordinary wave. We need to find the intersection of the ellipsoid $x^2\left(\frac{1}{n_o^2}+2p_{14}\bar{S}_{yz}\right)+y^2\left(\frac{1}{n_o^2}-2p_{14}\bar{S}_{yz}\right)=1$ and the vector $a\Big(\cos\Big(\sin^{-1}(\frac{\sin\phi}{n_o})\Big)\hat{a}_x-\frac{\sin\phi\tan\theta\cos\psi}{n_o\sqrt{\tan^2\theta\cos^2\psi+1}}\hat{a}_y\Big)$. This vector is a scaled form of $\bar{p}_o$ with the scalar a, and the length of this vector is equal to the refractive index $n_o(t)$ experienced by the ordinary wave. Using the intersection of the ellipsoid and the vector, we arrive at: $a^2\cos^2\Big(\sin^{-1}(\frac{\sin\phi}{n_o})\Big)\left(\frac{1}{n_o^2}+2p_{14}\bar{S}_{yz}\right)+\frac{a^2\sin^2\phi\tan^2\theta\cos^2\psi}{n_o^2(\tan^2\theta\cos^2\psi+1)}\left(\frac{1}{n_o^2}-2p_{14}\bar{S}_{yz}\right)=1$. The length of the scaled vector can be found as:

$$n_o^2(t)=\frac{n_o^2}{1+\frac{2p_{14}\bar{S}_{yz}n_o^2(n_o^2\cos^2(\sin^{-1}(\frac{\sin\phi}{n_o}))(\tan^2\theta\cos^2\psi+1))-\sin^2\phi\tan^2\theta\cos^2\psi}{n_o^2(\tan^2\theta\cos^2\psi+1)\cos^2(\sin^{-1}(\frac{\sin\phi}{n_o}))+\sin^2\phi\tan^2\theta\cos^2\psi}} \tag{S29}$$

$$n_o(t)\approx n_o\left(1-\frac{p_{14}\bar{S}_{yz}n_o^2\Big(n_o^2\cos^2(\sin^{-1}(\frac{\sin\phi}{n_o}))(\tan^2\theta\cos^2\psi+1)-\sin^2\phi\tan^2\theta\cos^2\psi\Big)}{n_o^2(\tan^2\theta\cos^2\psi+1)\cos^2(\sin^{-1}(\frac{\sin\phi}{n_o}))+\sin^2\phi\tan^2\theta\cos^2\psi}\right) \tag{S30}$$

We now solve for the extraordinary wave. We need to find the intersection of the ellipsoid $x^2\left(\frac{1}{n_o^2}+2p_{14}\bar{S}_{yz}\right)+y^2\left(\frac{1}{n_o^2}-2p_{14}\bar{S}_{yz}\right)+z^2\left(\frac{1}{n_e^2}\right)=1$ and the vector $b\big(-\cos^2\theta_e\tan\theta\cos\psi\hat{a}_x-\cos\tilde{\theta}_e\cos\theta_e\hat{a}_y+(\cos^2\tilde{\theta}_e+\cos^2\theta_e\tan^2\theta\cos^2\psi)\hat{a}_z\big)$. This vector is a scaled form of $\bar{p}_e$ with the scalar b. Using the intersection of the ellipsoid and the vector, we arrive at: $b^2(\cos^4\theta_e\tan^2\theta\cos^2\psi)(\frac{1}{n_o^2}+2p_{14}\bar{S}_{yz})+b^2\cos^2\tilde{\theta}_e(\frac{1}{n_o^2}-2p_{14}\bar{S}_{yz})+b^2(\cos^2\tilde{\theta}_e+\cos^2\theta_e\tan^2\theta\cos^2\psi)^2(\frac{1}{n_e^2})=1$. The length of the scaled vector can be found as:

$$n_e^2(t)=\frac{n_o^2n_e^2}{n_o^2\sin^2\theta_e+n_e^2\cos^2\theta_e+\frac{2p_{14}\bar{S}_{yz}n_o^2(n_e^2\cos^4\theta_e\tan^2\theta\cos^2\psi-\cos^2\tilde{\theta}_e\cos^2\theta_e n_e^2)}{\cos^4\theta_e\tan^2\theta\cos^2\psi+\cos^2\tilde{\theta}_e\cos^2\theta_e+(\cos^2\tilde{\theta}_e+\cos^2\theta_e\tan^2\theta\cos^2\psi)^2}} \tag{S31}$$

$$n_e(t)\approx\frac{n_on_e}{\sqrt{n_o^2\sin^2\theta_e+n_e^2\cos^2\theta_e}}\left(1-\frac{p_{14}\bar{S}_{yz}n_o^2\big(n_e^2\cos^4\theta_e\tan^2\theta\cos^2\psi-\cos^2\tilde{\theta}_e\cos^2\theta_e n_e^2\big)}{(n_o^2\sin^2\theta_e+n_e^2\cos^2\theta_e)(\cos^4\theta_e\tan^2\theta\cos^2\psi+\cos^2\tilde{\theta}_e\cos^2\theta_e+(\cos^2\tilde{\theta}_e+\cos^2\theta_e\tan^2\theta\cos^2\psi)^2)}\right) \tag{S32}$$

We can now calculate the time-varying phase picked up by the ordinary and extraordinary waves after propagating through the wafer as $\phi_o(t)$ and $\phi_e(t)$, respectively. These are expressed as follows:

$$\phi_o(t)=\frac{2\pi L}{\lambda}\cos(\tilde{\theta}_o)n_o(t) \tag{S33}$$

$$\phi_e(t)=\frac{2\pi L}{\lambda}\cos(\tilde{\theta}_e)n_e(t) \tag{S34}$$

For the expressions above, λ is the free-space wavelength of the plane wave and L is the thickness of the wafer (parallel to $\hat{a}_y$). We can express the static (ϕ_s) and dynamic (ϕ_D) phase difference as:

$$\phi_s+\phi_D\cos(2\pi f_ct)=\phi_o(t)-\phi_e(t) \tag{S35}$$

We will now turn our attention to the second component, the second polarizer (analyzer). The electric field incident on the second polarizer is given as:

$$\bar{E}_1(t)=\sqrt{\frac{I_0}{2}}\Big(\hat{p}_{oi}c_oe^{j(w_Lt+\phi_o(t))}+\hat{p}_{ei}c_ee^{j(w_Lt+\phi_e(t))}\Big) \tag{S36}$$

In the equation above, w_L is the angular frequency of the optical field. After propagating through the second polarizer, the electric field becomes:

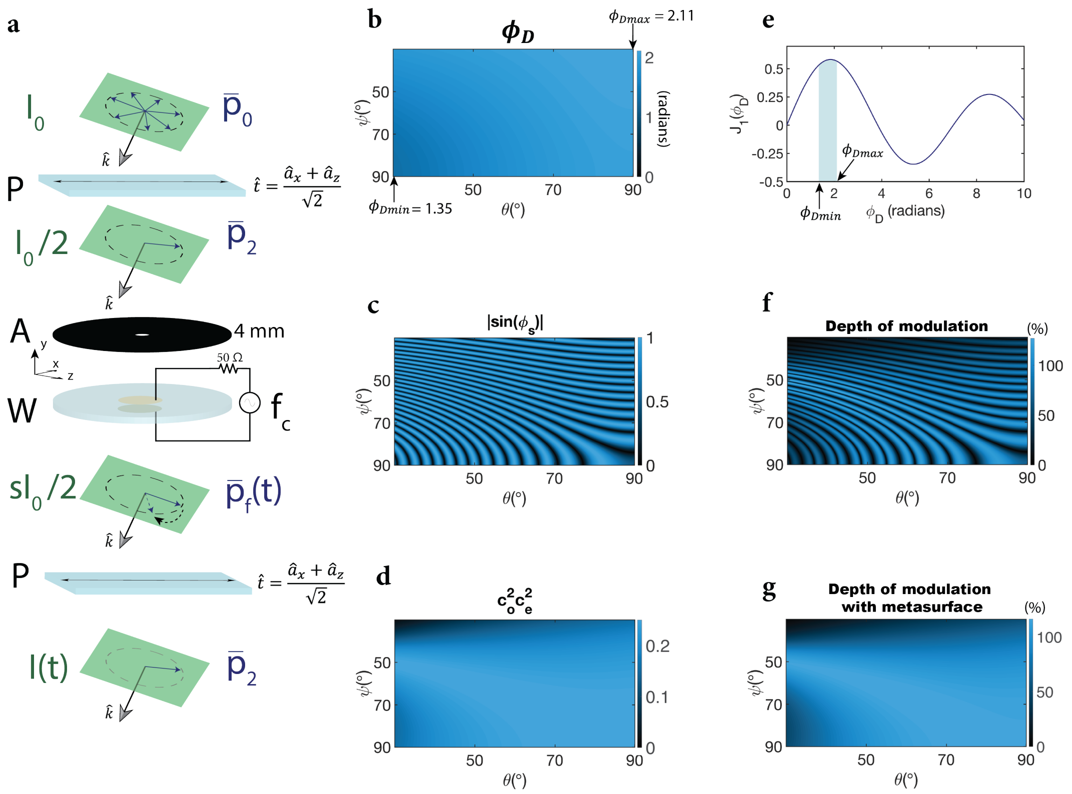

Fig. S3. Intensity modulation of a plane wave for optimal strain in the wafer. **a** The intensity modulation for a plane wave that is incident on the intensity modulator with wave vector $\hat{k}$ is shown. Standard spherical coordinate notation is used for the angles to define $\hat{k} = \hat{a}_x \sin\theta\cos\psi + \hat{a}_y \sin\theta\sin\psi + \hat{a}_z\cos\theta$. The plane wave has random polarization $\bar{p}_0$ and intensity I_0. After passing through the first polarizer (P) with transmission axis $\hat{t} = (\hat{a}_x + \hat{a}_z)/\sqrt{2}$, the intensity of the plane wave is reduced to $I_0/2$, with a polarization direction of $\bar{p}_2$. The plane wave then passes through an aperture (A) with an aperture diameter of 4 mm, so that only the center part of the S_{yz} strain profile is used. The plane wave then passes through the wafer (W) that has a volume average strain $\bar{S}_{yz} = 5.67 \times 10^{-4}$ at $f_c = 3.7696$ MHz. The plane wave that has propagated through the wafer has intensity reduced to $sI_0/2$, with time-dependent polarization $\bar{p}_f(t)$. $0 < s < 1$ captures the attenuation of the plane wave due to passing through the aperture. The polarization rotation of the plane wave is converted into intensity modulation after passing through the second polarizer (P). The intensity of the plane wave $I(t)$ is now time-dependent and has a polarization direction of $\bar{p}_2$. **b** The dynamic phase accumulated (ϕ_D) by plane waves incident at different angles to the wafer is shown. The minimum and maximum values attained by ϕ_D are $\phi_{Dmin} = 1.35$ and $\phi_{Dmax} = 2.11$, respectively. **c** $|\sin(\phi_s)|$ for plane waves incident at different angles to the wafer is shown, where ϕ_s is the static phase accumulated by the plane waves. **d** $c_o^2 c_e^2$ for plane waves incident at different angles to the wafer is shown, where c_o is the amplitude of the excited ordinary wave, and c_e is the amplitude of the excited extraordinary wave. **e** $J_1(\phi_D)$ is shown as a function of ϕ_D. The maximum and minimum for $J_1(\phi_D)$ corresponding to the maximum and minimum ϕ_D in **(b)** is shown. **f** Depth of modulation (DoM) as a percentage is shown for plane waves incident on the wafer at different angles. **g** Depth of modulation (DoM) as a percentage is shown for plane waves incident on the wafer at different angles. An ideal polarization manipulating metasurface is assumed to be coated on the wafer surface such that $\sin\phi_s = 1 \; \forall \; (\theta, \psi)$.

$$\bar{E}_2(t) = \sqrt{\frac{I_0}{2}}\Big((\hat{p}_{oi} \cdot \hat{p}_2)\hat{p}_2 c_o e^{j(w_L t + \phi_o(t))} + (\hat{p}_{ei} \cdot \hat{p}_2)\hat{p}_2 c_e e^{j(w_L t + \phi_e(t))}\Big) = \sqrt{\frac{I_0}{2}}\Big(\hat{p}_2 c_o^2 e^{j(w_L t + \phi_o(t))} + \hat{p}_2 c_e^2 e^{j(w_L t + \phi_e(t))}\Big) \tag{S37}$$

The intensity of the plane wave that has propagated through the second polarizer is given as follows, which is written in terms of the harmonics of f_c using the Jacobi-Anger expansion:

$$I(t) = \left|\bar{E}_2(t)\right|^2 = \frac{I_0}{2}\Big(c_o^4 + c_e^4 + 2c_o^2 c_e^2 \cos(\phi_s + \phi_D \cos(2\pi f_c t))\Big)$$
$$= \frac{I_0}{2}\left(c_o^4 + c_e^4 + 2c_o^2 c_e^2\left(\cos\phi_s\Big(J_0(\phi_D) + 2\sum_{n=1}^{\infty}(-1)^n J_{2n}(\phi_D)\cos(4\pi f_c t)\Big) + 2\sin\phi_s\Big(\sum_{n=1}^{\infty}(-1)^n J_{2n-1}(\phi_D)\cos((2n-1)2\pi f_c t)\Big)\right)\right) \tag{S38}$$

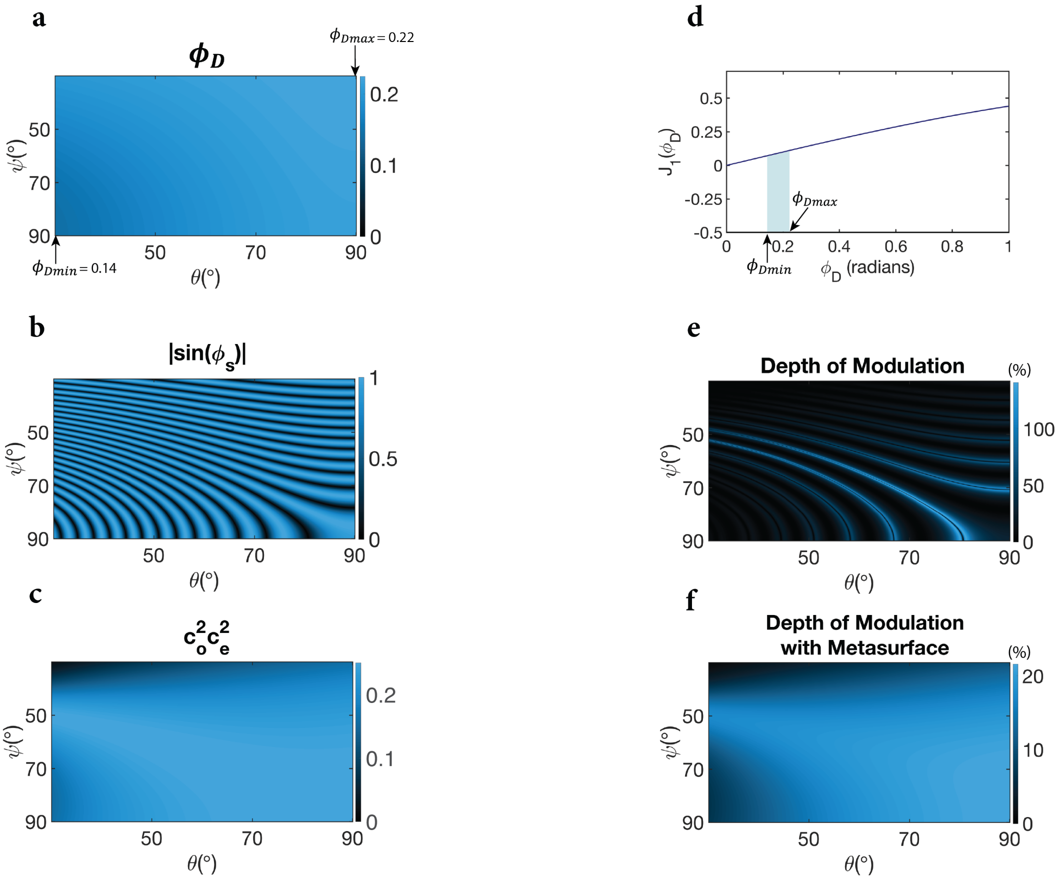

Fig. S4. Intensity modulation of a plane wave. Volume average strain $\bar{S}_{yz} = 6 \times 10^{-5}$ at $f_c = 3.7696$ MHz is used for this figure. This volume average strain is computed for a diameter of 4 mm centered on the wafer. **a** The dynamic phase accumulated (ϕ_D) by plane waves incident at different angles to the wafer is shown. The minimum and maximum values attained by ϕ_D are $\phi_{Dmin} = 0.14$ and $\phi_{Dmax} = 0.22$, respectively. **b** $|\sin(\phi_s)|$ for plane waves incident at different angles to the wafer is shown, where ϕ_s is the static phase accumulated by the plane waves. **c** $c_o^2 c_e^2$ for plane waves incident at different angles to the wafer is shown, where c_o is the amplitude of the excited ordinary wave, and c_e is the amplitude of the excited extraordinary wave. **d** $J_1(\phi_D)$ is shown as a function of ϕ_D. The maximum and minimum for $J_1(\phi_D)$ corresponding to the maximum and minimum ϕ_D in **a** is shown. **e** Depth of modulation (DoM) as a percentage is shown for plane waves incident on the wafer at different angles. **f** Depth of modulation (DoM) as a percentage is shown for plane waves incident on the wafer at different angles. An ideal polarization manipulating metasurface is assumed to be coated on the wafer surface such that $\sin\phi_s = 1 \ \forall \ (\theta, \psi)$.

Now we will look into the intensity modulation term P_{f_c} at frequency f_c, and the constant term (DC). These are expressed as follows:

$$\begin{aligned} \mathrm{DC} &= \frac{I_0}{2}\left(c_o^4 + c_e^4 + 2c_o^2c_e^2 J_0(\phi_D)\cos\phi_s\right) \\ P_{f_c} &= 2I_0 c_o^2 c_e^2 J_1(\phi_D)\sin\phi_s \end{aligned} \tag{S39}$$

We can define the depth of modulation (DoM) as:

$$\mathrm{DoM} = \frac{4c_o^2c_e^2 J_1(\phi_D)\sin\phi_s}{c_o^4 + c_e^4 + 2c_o^2c_e^2 J_0(\phi_D)\cos\phi_s} \tag{S40}$$

There are two important observations to make regarding equation (S39) and equation (S40). First, P_{f_c} can be greater than $\frac{I_0}{4}$ since $J_1(\phi_D)$ can exceed 0.5. This means that for the same peak-to-peak variation as a pure sinusoid at frequency f_c, the power in the fundamental of the Bessel function is larger. This is similar to how a square wave has a fundamental tone larger than a sinusoid with the same frequency and peak-to-peak variation.

The second observation is that even if ϕ_D is small so that $P_{fc} << \frac{I_0}{4}$, it is still possible for DoM > 1. The reason why this can happen is because the DC value is affected by both ϕ_s and ϕ_D. Therefore, it can be that DC ≈ 0, leading to DoM > 1 even though $P_{fc} << \frac{I_0}{4}$.

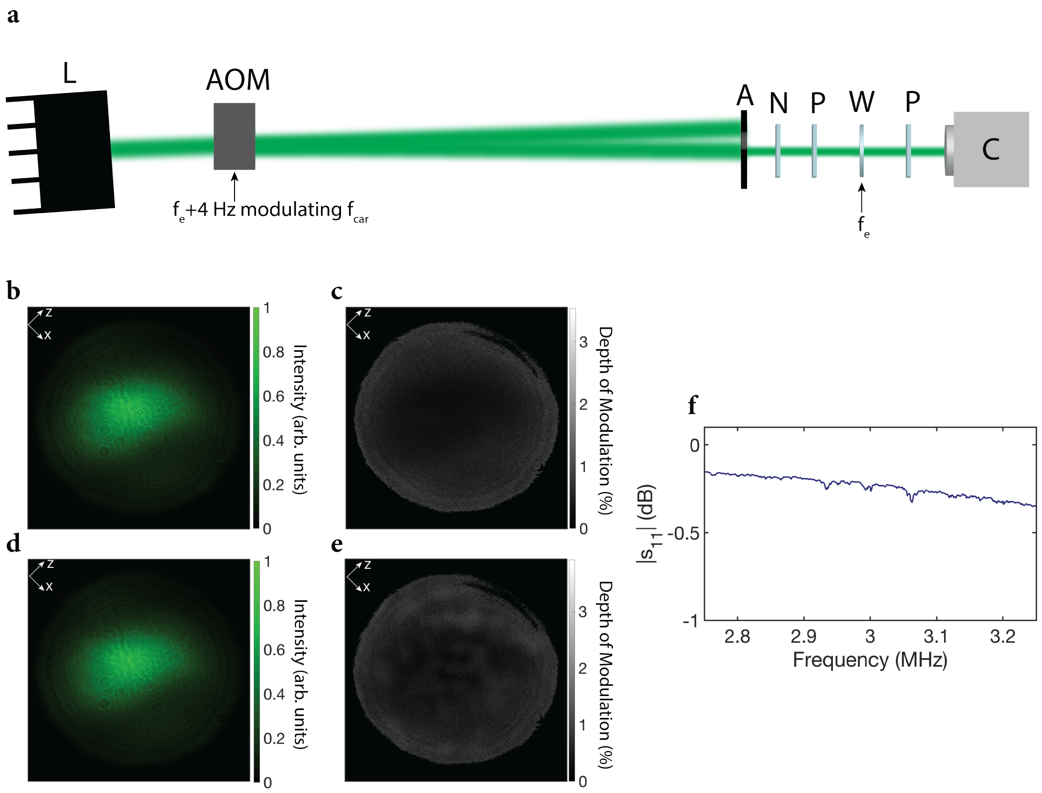

Fig. S5. Checking contribution of electro-optic effect. **a** Schematic of the characterization setup is shown. The setup includes a laser (L) with a wavelength of 532 nm that passes through a free-space acousto-optic modulator (AOM). The laser beam is intensity modulated at $f_e + 4$ Hz = 3.000004 MHz by modulating the carrier frequency f_{car} = 80 MHz exciting the AOM. The setup also includes an aperture (A) with a diameter of 9.3 mm, a neutral density filter (N), two polarizers (P) with transmission axis $\hat{t}$, wafer (W), and a standard CMOS camera (C). The camera detects the intensity modulated laser beam with a frame rate of 30 Hz. **b** Time-averaged intensity profile of the laser beam detected by the camera is shown for $\phi = 0$ and when no voltage is applied to wafer surface electrodes. **c** The DoM at 4 Hz of the laser beam is shown per pixel for $\phi = 0$ and when no voltage is applied to wafer surface electrodes. **d** Time-averaged intensity profile of the laser beam detected by the camera is shown for $\phi = 0$ and when 90 mW of RF power at $f_e = 3$ MHz is applied to wafer surface electrodes. **e** The DoM at 4 Hz of the laser beam is shown per pixel for $\phi = 0$ and when 90 mW of RF power at $f_e = 3$ MHz is applied to wafer surface electrodes. **f** s_{11} scan with respect to 50 Ω and using 0 dBm excitation power with a bandwidth of 100 Hz is shown around 3 MHz.

Fig. S3 and Fig. S4 show the plots of how the variables that influence $I(t)$ vary as a function of the incoming angle of the plane wave (θ, ψ). For Fig. S3 and Fig. S4, $L = 505$ μm ($\frac{3.7696 \text{ MHz}}{3.7337 \text{ MHz}} \times 500$ μm ≈ 505 μm), $n_o = 2.2965$, $n_e = 2.215$. These variables are chosen to be consistent with experimental measurements in the main text. Notice that even though most points on the DoM for Fig. S4 are small, there are specific angles for which DoM > 1 due to the explanation in the previous paragraph.

3 Contribution of Electro-Optic Effect

The linear electro-optic effect can also modulate the polarization of light in lithium niobate. The modified index ellipsoid when the electric field E is applied along the y direction through the surface electrodes is as follows:

$$\left(\frac{1}{n_o^2} - r_{22}E\right)x^2 + \left(\frac{1}{n_o^2} + r_{22}E\right)y^2 + \left(\frac{1}{n_e^2}\right)z^2 + 2yzr_{51}E = 1 \tag{S41}$$

We now apply a rotation to the yz axis such the the new form is diagonal (as was carried out for the strain tensor in Section 1). Using the coordinate transformation in equation (S3), equation (S41) can be transformed into:

$$\Big(\frac{1}{n_o^2} - r_{22}E\Big)x^2 + \Big(\frac{1}{n_o^2} + r_{22}E + r_{51}E\tan\theta\Big)y'^2 + \Big(\frac{1}{n_e^2} - r_{51}E\tan\theta\Big)z'^2 = 1$$
$$\tan(2\theta) = \frac{2r_{51}E}{\frac{1}{n_o^2} + r_{22}E - \frac{1}{n_e^2}} \tag{S42}$$

Since $\tan\theta \ll 1$, we neglect the modulations of the y' and z' axis which include the $\tan\theta$ term. The modified index ellipsoid can therefore be approximated as:

$$\Big(\frac{1}{n_o^2} - r_{22}E\Big)x^2 + \Big(\frac{1}{n_o^2} + r_{22}E\Big)y'^2 + \Big(\frac{1}{n_e^2}\Big)z'^2 = 1 \tag{S43}$$

Using the electro-optic coefficient of $r_{22} = 6.7 \times 10^{-12}$ m/V and an electric field of $E = 2 \times 10^3$ V/m (2Vpp applied to wafer electrodes), time-varying birefringence induced by the linear electro-optic effect is approximately $\Delta n_{eo} = 0.5En_o^3r_{22} \approx 8.2 \times 10^{-8}$. For the same simulation, $\bar{S}_{yz} = 1.00 \times 10^{-5}$ with $p_{14} = 0.05$. The time-varying birefringence induced by the photoelastic effect is approximately $\Delta n_{pe} = n_o^3 p_{14}\bar{S}_{yz} \approx 6.1 \times 10^{-6}$.

$$\frac{\Delta n_{eo}}{\Delta n_{pe}} \approx 0.01 \tag{S44}$$

We see that the photoelastic effect is approximately two orders of magnitude stronger than the linear electro-optic effect. Therefore, the contribution of the linear electro-optic effect can be neglected. We carry out a measurement to verify this finding. We measure the contribution of the linear electro-optic effect off resonance at 3 MHz. The measurement setup and the results are shown in Fig. S5. It is clear that the electro-optic effect has a negligible contribution.

4 Intensity Modulation Efficiency

In this section, we will calculate the modulation efficiency of the intensity modulator experimentally, and extract the volume average strain in the wafer. The measurement setup in Fig. S6**a** is used. For measurements shown in Fig. S6**b,c,d**, the second polarizer is inserted between the wafer (W) and the camera (C), but no voltage is applied to the wafer surface electrodes. This is a control measurement to make sure no modulation is present on the laser beam without voltage applied to the electrodes.

Fig. S6**e,f,g** show the measurements when 90 mW of RF power is applied to the wafer surface electrodes, but the second polarizer is removed. This measurement is done to show that the dominant intensity modulation effect manifests itself when the second polarizer is present (and therefore intensity modulation is caused via polarization modulation). The intensity modulation seen in Fig. S6**f** without the second polarizer is likely due to anisotropic Bragg diffraction[3,4], where some portion of the laser beam is scattered and Doppler shifted by $f_r = 3.7337$ MHz. The camera detects the beating between the optical pump beam and the scattered and Doppler shifted beam, which appears as intensity modulation.

Fig. S6**h,i,j** show the measurements when 90 mW of RF power is applied to the wafer surface electrodes, and the second polarizer is inserted between the wafer (W) and the camera (C). It is clear that the dominant intensity modulation mechanism is polarization modulation when we compare Fig. S6**i** to Fig. S6**f**. Using Fig. S6**e,h,i**, we can calculate $|\sin\phi_s|$ and ϕ_D. Without the second polarizer, the camera detects $\frac{I_0}{2}$. When the second polarizer is inserted, the DC value is: $\frac{I_0}{2}\big(c_o^4 + c_e^4 + 2c_o^2c_e^2\cos\phi_s J_0(\phi_D)\big)$. Since the laser beam is incident at a normal angle to the wafer, $c_o = c_e = \frac{1}{\sqrt{2}}$. We take the ratio of the sum of pixels in Fig. S6**h** to Fig. S6**e**, which yields approximately 0.47. To simplify the calculations, we assume it is 0.5. For this ratio to be 0.5, $\cos\phi_s = 0$. This means $|\sin\phi_s| = 1$. The modulation power at frequency f_r can now be expressed as $\frac{I_0 J_1(\phi_D)}{2}$. We can estimate $J_1(\phi_D)$ by taking the ratio of the pixels in Fig. S6**i** to Fig. S6**e**. Since we know that $\phi_D < 1$, we use the following approximation: $J_1(\phi_D) \approx \frac{\phi_D}{2}$. The average across all pixels for ϕ_D is $\bar{\phi}_D \approx 0.15$. We now estimate the volume average strain in the wafer as:

$$\bar{S}_{yz} = \frac{\bar{\phi}_D\lambda}{2\pi L p_{14} n_o^3} \approx 4.15 \times 10^{-5} \tag{S45}$$

The reason behind using the peak-to-peak variation in Fig. S6 is to compensate for the heterodyne detection. The intensity modulation imparted on the laser beam by the modulator generates a beat tone at 4 Hz, but at half the power.

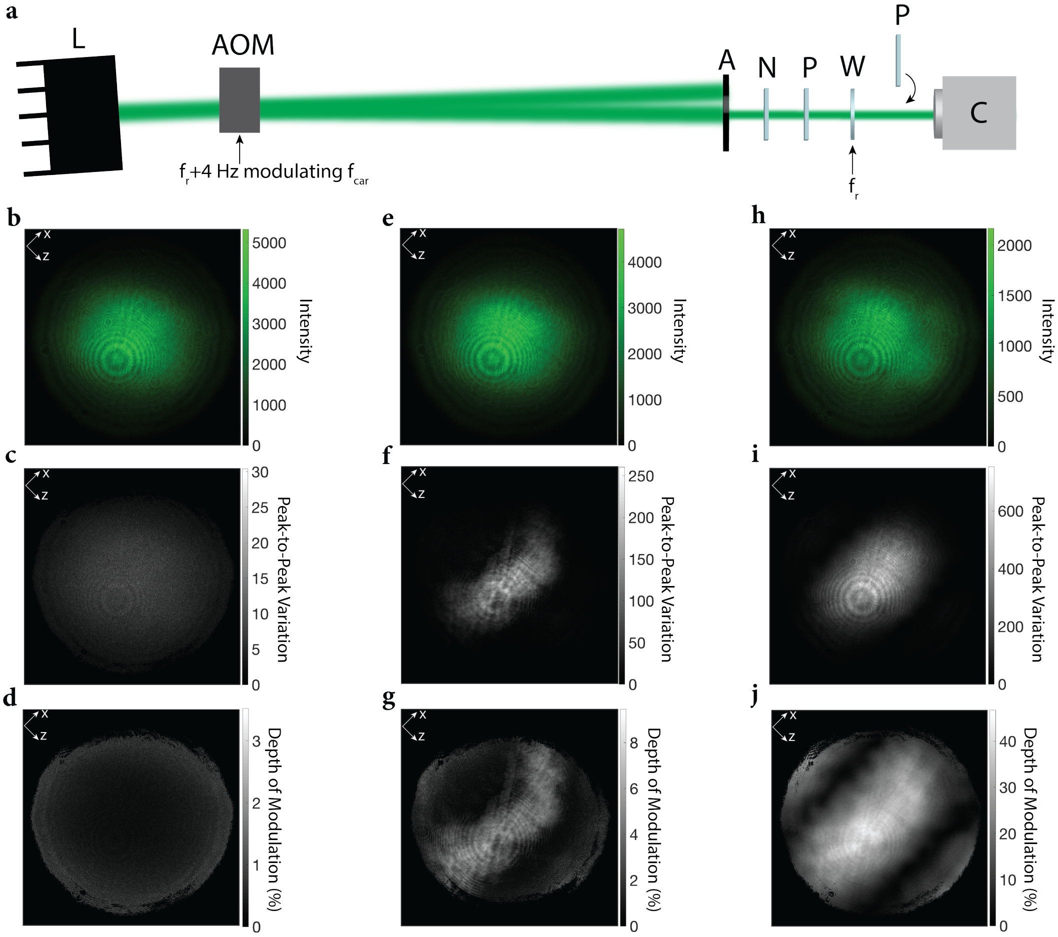

Fig. S6. Measurement of modulation efficiency. **a** Schematic of the characterization setup is shown. The setup includes a laser (L) with a wavelength of 532 nm that passes through a free-space acousto-optic modulator (AOM). The laser beam is intensity modulated at $f_r + 4$ Hz $= 3.733704$ MHz by modulating the carrier frequency $f_{car} = 80$ MHz exciting the AOM. The setup also includes an aperture (A) with a diameter of 1 cm, a neutral density filter (N), two polarizers (P) with transmission axis $\hat{t}$, wafer (W), and a standard CMOS camera (C). The camera detects the intensity modulated laser beam with a frame rate of 30 Hz. **b** Time-averaged intensity profile of the laser beam detected by the camera is shown for $\phi = 0$ and when no voltage is applied to wafer surface electrodes. The second polarizer (P) is inserted between (W) and (C) for this measurement. **c** The peak-to-peak variation at 4 Hz of the laser beam is shown per pixel for $\phi = 0$ and when no voltage is applied to wafer surface electrodes. The second polarizer (P) is inserted between (W) and (C) for this measurement. **d** The DoM at 4 Hz of the laser beam is shown per pixel for $\phi = 0$ and when no voltage is applied to wafer surface electrodes. The second polarizer (P) is inserted between (W) and (C) for this measurement. **e** Time-averaged intensity profile of the laser beam detected by the camera is shown for $\phi = 0$ and when 90 mW of RF power at f_r is applied to wafer surface electrodes. The second polarizer (P) is not inserted between (W) and (C) for this measurement. **f** The peak-to-peak variation at 4 Hz of the laser beam is shown per pixel for $\phi = 0$ and when 90 mW of RF power at f_r is applied to wafer surface electrodes. The second polarizer (P) is not inserted between (W) and (C) for this measurement. **g** The DoM at 4 Hz of the laser beam is shown per pixel for $\phi = 0$ and when 90 mW of RF power at f_r is applied to wafer surface electrodes. The second polarizer (P) is not inserted between (W) and (C) for this measurement. **h** Time-averaged intensity profile of the laser beam detected by the camera is shown for $\phi = 0$ and when 90 mW of RF power at f_r is applied to wafer surface electrodes. The second polarizer (P) is inserted between (W) and (C) for this measurement. **i** The peak-to-peak variation at 4 Hz of the laser beam is shown per pixel for $\phi = 0$ and when 90 mW of RF power at f_r is applied to wafer surface electrodes. The second polarizer (P) is inserted between (W) and (C) for this measurement. **j** The DoM at 4 Hz of the laser beam is shown per pixel for $\phi = 0$ and when 90 mW of RF power at f_r is applied to wafer surface electrodes. The second polarizer (P) is inserted between (W) and (C) for this measurement.

5 s_{11} Measurement at Different RF Power Levels

In this section, we show how the s_{11} properties of the modulator change when excited at different RF power levels. The s_{11} is measured with 0 dBm power using a VNA (vector network analyzer), and the magnitude and phase plots

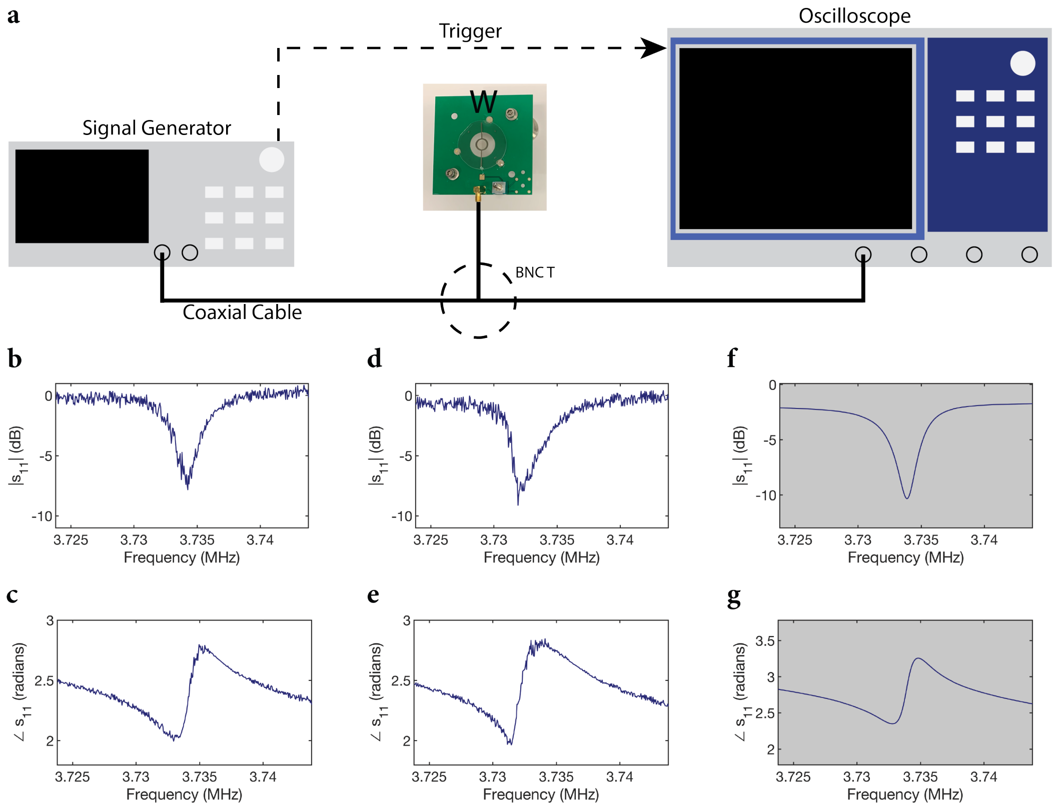

Fig. S7. Measuring s_{11} at different excitation powers. **a** Schematic of the characterization setup is shown. The setup includes a signal generator exciting the wafer (W) surface electrodes and triggering an oscilloscope. The oscilloscope detects the voltage on the wafer surface electrodes with high input impedance (1 MΩ). **b** Computed $|s_{11}|$ using the setup shown in **(a)** with respect to 50 Ω using 4.0 dBm excitation power with a bandwidth of 1 Hz for the desired acoustic mode. **c** Computed $\angle s_{11}$ using the setup shown in **(a)** with respect to 50 Ω using 4.0 dBm excitation power with a bandwidth of 1 Hz for the desired acoustic mode. **d** Computed $|s_{11}|$ using the setup shown in **(a)** with respect to 50 Ω using 19.5 dBm excitation power with a bandwidth of 1 Hz for the desired acoustic mode. **e** Computed $\angle s_{11}$ using the setup shown in **(a)** with respect to 50 Ω using 19.5 dBm excitation power with a bandwidth of 1 Hz for the desired acoustic mode. **f** $|s_{11}|$ scan using a VNA with respect to 50 Ω with 0 dBm excitation power and with a bandwidth of 20 Hz for the desired acoustic mode. **g** $\angle s_{11}$ scan using a VNA with respect to 50 Ω with 0 dBm excitation power and with a bandwidth of 20 Hz for the desired acoustic mode.

are shown in Fig. S7**f,g**. Since optical characterization of the modulator is performed at a higher RF power (90 mW = 19.5 dBm) to achieve higher modulation efficiencies, we also need to measure the s_{11} at this RF power level. The setup shown in Fig. S7**a** is used for this measurement, where a signal generator allows us to measure at power levels higher than possible with the VNA. We see that for an excitation power of 4.0 dBm, we get a very similar response to what was measured using the VNA, as shown in Fig. S7**b,c**. This confirms that the measurement setup is consistent with the VNA measurements. Next we perform an s_{11} measurement with 19.5 dBm of RF power; the RF power used for optical characterization. We see a shift in the resonance frequency of approximately 2 kHz in Fig. S7**d,e**. This shift is most likely caused by a change in the lithium niobate temperature due to the higher excitation power. For a frequency shift of 2 kHz, the excited volume average strain level in COMSOL shows negligible change.

6 Intensity Modulation Efficiency of Different Modulation Mechanisms

In this section, we will calculate the modulation efficiency of the proposed intensity modulator and other modulators relying on different modulation mechanisms. We first begin by calculating the modulation efficiency for the approach discussed in this paper: resonant photoelastic modulation. The modulator has a thickness of 0.5 mm and a usable aperture diameter of 1 cm. The volume average dynamic phase is $\bar{\phi}_D = 0.15$ for RF excitation power of 90 mW. For the first order Bessel function of the first kind to be equal to 0.5, the volume average dynamic phase should be 1.2. We can now calculate the modulation efficiency by computing the required RF power to switch the photoelastic modulator at 3.7 MHz, at a wavelength of 532 nm, and with an input aperture of 1 cm^2:

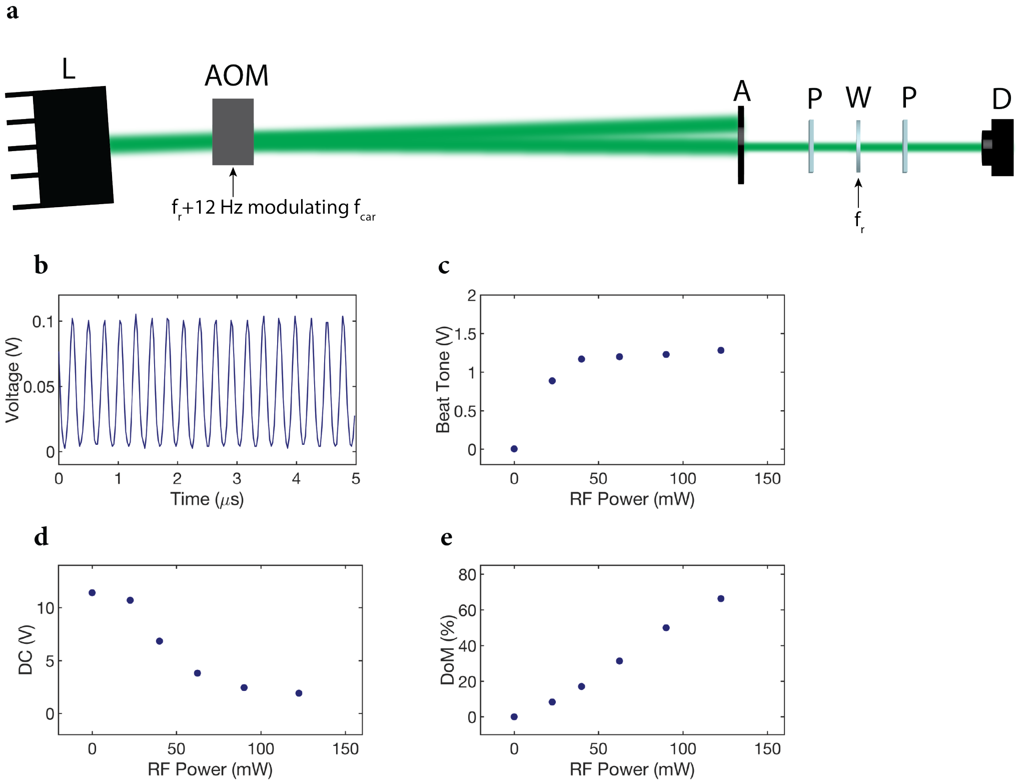

Fig. S8. Depth of modulation measurement at different RF excitation power levels. **a** Schematic of the characterization setup is shown. The setup includes a laser (L) with a wavelength of 532 nm that passes through a free-space acousto-optic modulator (AOM). The laser beam is intensity modulated at $f_r + 12$ Hz $= 3.733712$ MHz by modulating the carrier frequency $f_{car} = 80$ MHz exciting the AOM. The setup also includes an aperture (A) with a diameter of 2.5 mm, two polarizers (P) with transmission axis $\hat{t}$, wafer (W), and a free-space photodetector (D). The photodetector detects the intensity modulated laser beam with sampling rate of 40 MHz. **b** Measured intensity modulation profile for the laser beam using the photodetector is shown when no voltage is applied to wafer surface electrodes. **c** The peak-to-peak variation for the beat tone at 12 Hz is shown when the wafer surface electrodes are excited at different RF power levels. **d** The DC level for the intensity of the laser beam is shown when the wafer surface electrodes are excited at different RF power levels. **e** The DoM at 12 Hz for the laser beam is shown when the wafer surface electrodes are excited at different RF power levels.

$$\mathrm{P}_{on*} = \left(\frac{1\ \mathrm{cm}^2}{\pi \times 0.5^2\ \mathrm{cm}^2}\right) \times \left(\frac{1.2}{0.15}\right)^2 \times 0.09\ \mathrm{W} \approx 7.4\ \mathrm{W} \tag{S46}$$

We now calculate for a commercial transverse resonant Pockels cell[5]. The Pockels cell has a thickness of 57.2 mm and an aperture diameter of 2 mm. The half-wave voltage at a wavelength of 633 nm is 15 V. To make a fair comparison to our modulator and have the same temperature tolerance, we assume a thickness of 0.5 mm for the Pockels cell. We can now calculate the modulation efficiency by computing the required RF power to switch the Pockels cell at 3.7 MHz, at a wavelength of 532 nm, and with an input aperture of 1 cm^2 (3 dB cutoff frequency due to RC time constant is not taken into account):

$$\mathrm{P}_{on1} = \frac{\left(\frac{15\ \mathrm{V}}{\sqrt{2}} \times \frac{532\ \mathrm{nm}}{633\ \mathrm{nm}} \times \frac{11.3\ \mathrm{mm}}{2\ \mathrm{mm}} \times \frac{57.2\ \mathrm{mm}}{0.5\ \mathrm{mm}}\right)^2}{50\ \Omega} \approx 6.7 \times 10^5\ \mathrm{W} \tag{S47}$$

We now calculate for a commercial longitudinal Pockels cell[6]. The Pockels cell has an aperture diameter of 9 mm and a thickness of 36 mm. The capacitance is 8 pF and the half-wave voltage is 3.3 kV. To make a fair comparison to our modulator and have the same temperature tolerance, we assume a thickness of 0.5 mm for the Pockels cell. We can now calculate the modulation efficiency by computing the required RF power to switch the Pockels cell at 3.7 MHz, at a wavelength of 532 nm, and with an input aperture of 1 cm^2 (3 dB cutoff frequency due to RC time constant is not taken into account):

$$\mathrm{P}_{on2} = \frac{1}{2}\left(\frac{11.3\ \mathrm{mm}}{9.0\ \mathrm{mm}}\right)^2 \times \left(3{,}300\ \mathrm{V}\right)^2 \times \frac{8 \times 10^{-12}\ \mathrm{F} \times 36\ \mathrm{mm}}{0.5\ \mathrm{mm}} \times 3.7 \times 10^6\ \mathrm{Hz} \approx 1.83 \times 10^4\ \mathrm{W} \tag{S48}$$

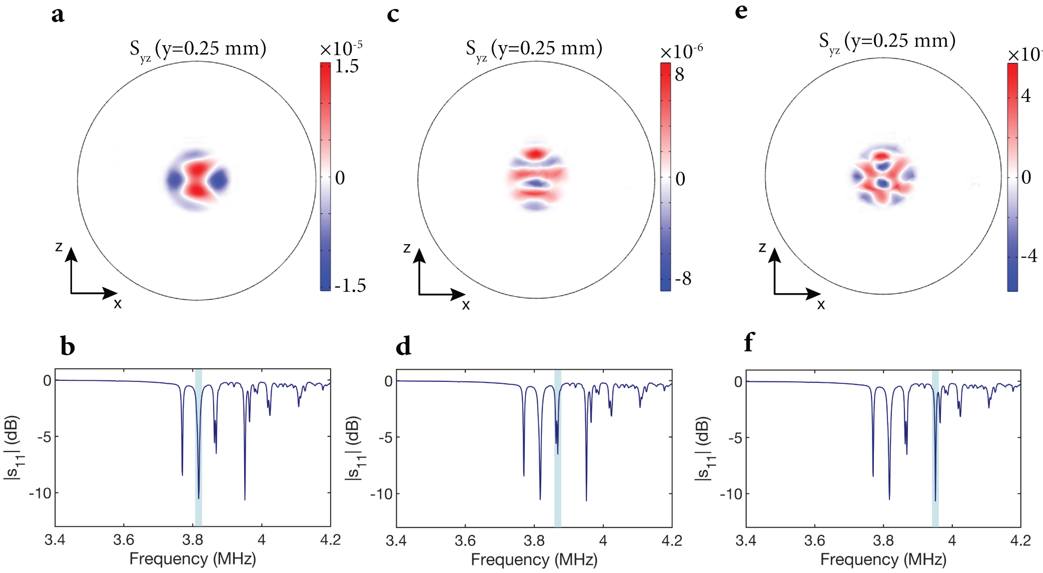

Fig. S9. Strain profile in the wafer for other acoustic modes. **a** S_{yz} strain profile for the plane that is parallel and 0.25 mm above the bottom wafer surface. 2Vpp is applied to wafer surface electrodes at a frequency of 3.817 MHz. **b** Simulated $|s_{11}|$ of the wafer with respect to 50 Ω, with the light blue region showing the excited mode in **(a)**. **c** S_{yz} strain profile for the plane that is parallel and 0.25 mm above the bottom wafer surface. 2Vpp is applied to wafer surface electrodes at a frequency of 3.868 MHz. **d** Simulated $|s_{11}|$ of the wafer with respect to 50 Ω, with the light blue region showing the excited mode in **(c)**. **e** S_{yz} strain profile for the plane that is parallel and 0.25 mm above the bottom wafer surface. 2Vpp is applied to wafer surface electrodes at a frequency of 3.951 MHz. **f** Simulated $|s_{11}|$ of the wafer with respect to 50 Ω, with the light blue region showing the excited mode in **(e)**.

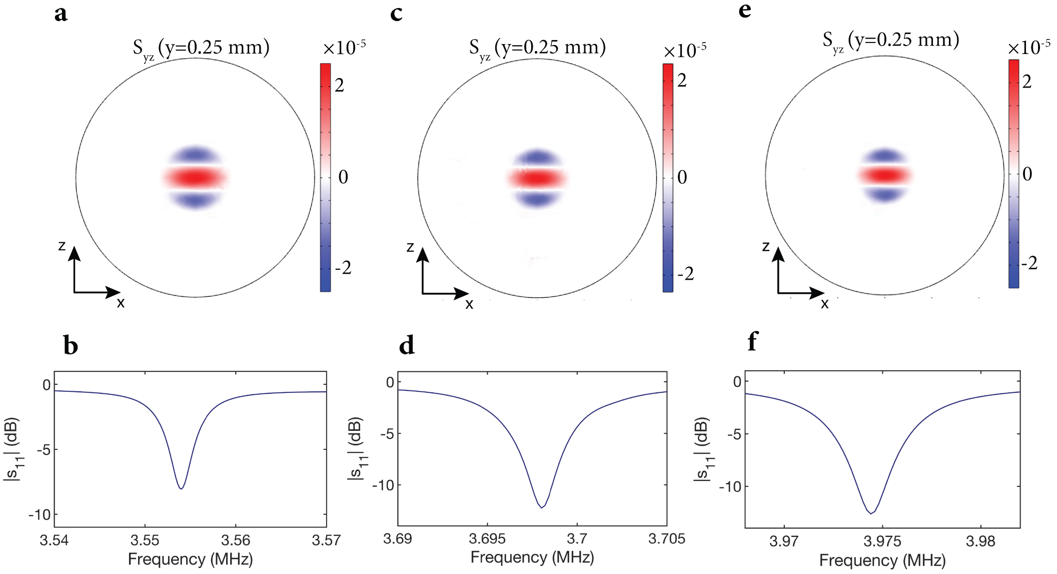

Fig. S10. Sensitivity analysis for the acoustic mode. **a** S_{yz} strain profile for the plane that is parallel and 0.25 mm above the bottom wafer surface. 2Vpp is applied to wafer surface electrodes at a frequency of 3.554 MHz. Wafer thickness is 530 μm, electrode diameter is 13.3 mm, and acoustic quality factor is 900. **b** Simulated $|s_{11}|$ of the wafer with respect to 50 Ω described in **(a)**. **c** S_{yz} strain profile for the plane that is parallel and 0.25 mm above the bottom wafer surface. 2Vpp is applied to wafer surface electrodes at a frequency of 3.698 MHz. Wafer thickness is 510 μm, electrode diameter is 12.3 mm, and acoustic quality factor is 1,100. **d** Simulated $|s_{11}|$ of the wafer with respect to 50 Ω described in **(c)**. **e** S_{yz} strain profile for the plane that is parallel and 0.25 mm above the bottom wafer surface. 2Vpp is applied to wafer surface electrodes at a frequency of 3.974 MHz. Wafer thickness is 475 μm, electrode diameter is 11.4 mm, and acoustic quality factor is 1,000. **f** Simulated $|s_{11}|$ of the wafer with respect to 50 Ω described in **(e)**.

We now calculate for an electroabsorption modulator relying on the quantum-confined Stark effect[7] for modulating the intensity of a laser beam. The modulator has aperture dimensions of 6 mm by 7 mm. The power

consumption is 14 W, yielding a depth of modulation of approximately 60%. The wavelength of operation is 860 nm. We can now calculate the modulation efficiency by computing the required RF power to switch the modulator at 3.7 MHz, with an input aperture of 1 cm^2 and assuming linear relationship between applied electric field and depth of intensity modulation (3 dB cutoff frequency due to RC time constant is not taken into account):

$$P_{on3} = 14\text{ W} \times \frac{3.7\text{ MHz}}{10\text{ MHz}} \times \left(\frac{100\%}{60\%}\right)^2 \times \left(\frac{1\text{ cm}^2}{0.6\text{ cm} \times 0.7\text{ cm}}\right) \approx 34\text{ W} \tag{S49}$$

We now calculate for a gate-tunable metasurface[8]. For this modulator, the unit area capacitance is 14 fF/μm^2, and 15% amplitude modulation is observed for an applied voltage of 2 V. The reported wavelength of operation is 1,550 nm. We can now calculate the modulation efficiency by computing the required RF power to switch the modulator at 3.7 MHz, with an input aperture of 1 cm^2 assuming linear relationship between applied electric field and intensity modulation efficiency (3 dB cutoff frequency due to RC time constant is not taken into account):

$$P_{on4} = \left(\frac{100\%}{15\%}\right)^2 \times \left(\frac{1}{2} \times 14 \times 10^{-15}\text{ F} \times (2\text{ V})^2 \times 3.7 \times 10^6\text{ Hz}\right) \times \left(\frac{1\text{ cm}}{1\ \mu\text{m}}\right)^2 \approx 460\text{ W} \tag{S50}$$

We now calculate for a plasmonic nanoresonator[9]. For this modulator, the switching energy is 283 fJ/μm^2, and the resulting amplitude modulation is 48%. The reported wavelength of operation is 1.34 μm. We can now calculate the modulation efficiency by computing the required RF power to switch the modulator at 3.7 MHz, with an input aperture of 1 cm^2 assuming linear relationship between applied electric field and intensity modulation efficiency (3 dB cutoff frequency due to RC time constant is not taken into account):

$$P_{on5} = 283 \times 10^{-15}\text{ J} \times \left(\frac{1\text{ cm}}{1\ \mu\text{m}}\right)^2 \times 3.7 \times 10^6\text{ Hz} \times \left(\frac{100\%}{48\%}\right)^2 \approx 454\text{ W} \tag{S51}$$

7 Strain Profile of Other Acoustic Modes

The dominant shear strain (S_{yz}) is shown for three other acoustic modes appearing around 4 MHz for the lithium niobate modulator in Fig. S9. Compared to the acoustic mode at 3.77 MHz, these modes have more variation (more null regions and anti-phase regions), making it more difficult to achieve uniform intensity modulation of a laser beam propagating through the active region of the wafer.

8 Tolerance of Acoustic Mode to Variations

The tolerance of the acoustic mode appearing around 3.77 MHz is investigated in this section. The variations considered for the modulator are: electrode diameter, wafer thickness, and acoustic quality factor. The results for different parameters are shown in Fig. S10. The acoustic mode is tolerant to small variations in these parameters (deviations by less than 10%). The acoustic resonance frequency varies with the wafer thickness; the electrical equivalent resistance for the acoustic mode varies with the electrode diameter and the acoustic quality factor.

9 Modulator Material Choice

The material of choice for the modulator is critical. The material should be chosen to have suitable piezoelectric and photoelastic tensors (based on the crystallographic point group of the material), as well as exhibit a strong acousto-optic figure of merit (AOFM).

Materials belonging to the trigonal crystal system with point group 3m allow strong coupling of RF to S_{yz} shear strain when the RF field is oriented along the y direction of the crystal (via the d_{15} piezoelectric tensor element). The excited S_{yz} shear strain couples to an optical beam via the photoelastic tensor element p_{14}. AOFM = $\frac{n^6 p_{14}^2}{\rho v^3}$, where n is the average of the ordinary and extraordinary refractive indices, p_{14} is the photoelastic tensor element, and v the acoustic velocity of S_{yz} in the material[10]. Lithium tantalate and lithium niobate belong to the trigonal crystal system with point group 3m, and are good candidates as a material for the modulator. They are optically transparent from visible and extending into the infrared wavelength range, have low acoustic loss, and are mass manufacturable with high purity at a low-cost. The AOFM for lithium tantalate and lithium niobate are 2.3×10^{-16} s^3kg^{-1} and 1.4×10^{-15} s^3kg^{-1}, respectively[2,11,12].

LN is chosen as the material for the modulator in this work since it has suitable piezoelectric and photoelastic tensors, a large optical transparency window, while offering high AOFM when compared to crystals belonging to the trigonal crystal system with point group 3m.

10 Reaching Higher Intensity Modulation Frequencies

The resonant frequency of the fundamental S_{yz} shear strain mode is inversely proportional to the wafer thickness to first order. Higher intensity modulation frequencies can be reached by using thinner LN wafers. If the wafer thickness is halved, the excited mode volume is also halved, but the average shear strain required to reach maximum intensity modulation contrast is doubled. The power in the shear strain S_{yz} is linearly proportional to the mode volume of the excited region multiplied by the average shear strain squared $\bar{S}_{yz}^2$. Therefore, the power required to excite the modulator for reaching maximum intensity modulation contrast is linearly proportional to the resonance frequency of the wafer to first order (assuming the acoustic quality factor remains the same for different thicknesses of the wafer).

To reduce the power consumption of the modulator operating at higher frequencies, the quality factor could be increased. However, if the cut angle of the wafer is not changed, this will result in the electrical equivalent resistance to decrease at the resonance frequency, making impedance matching difficult for small electrical equivalent resistances at resonance (especially for high quality factors). To increase the quality factor while keeping the electrical equivalent resistance matched to 50 Ω, a cut closer to the z axis of the wafer can be used.

Quality factor greater than 30,000 even at gigahertz frequencies is achievable for LN devices, with quality factor resonance frequency product of 10^{13} and higher[13,14]. Assuming Q = 30,000, COMSOL simulations are shown in Fig. S11 for different thicknesses to reach 11 MHz, 13 MHz, and 20 MHz resonance frequencies using a Y89 LN wafer. The electrical equivalent resistance is well matched to 50 Ω at resonance for these three thicknesses. The volume average shear strain $\bar{S}_{yz}$ over a 1 cm diameter region centered on the electrodes for the wafer that is 0.16 mm thick is 3.5×10^{-5}, when an RF excitation power of 6.9 mW is applied (2Vpp at resonance). The volume average shear strain $\bar{S}_{yz}$ over a 1 cm diameter region centered on the electrodes for the wafer that is 0.135 mm thick is 4.6×10^{-5}, when an RF excitation power of 11.9 mW is applied (2Vpp at resonance). The volume average shear strain $\bar{S}_{yz}$ over a 1 cm diameter region centered on the electrodes for the wafer that is 0.088 mm thick is 4.8×10^{-5}, when an RF excitation power of 12.6 mW is applied (2Vpp at resonance).

Maximum intensity modulation is reached when $\bar{\phi}_D = \frac{4\pi L p_{14} n_o^3 \bar{S}_{yz}}{\lambda} = 1.2$, where $\lambda = 532$ nm. For the wafer of thickness $L = 0.16$ mm, $\bar{S}_{yz} \approx 5.2 \times 10^{-4}$ to reach optimum modulation. This optimum strain level is reached if 6.9 mW $\times \left(\frac{5.2\times10^{-4}}{3.5\times10^{-5}}\right)^2 \approx 1.5$ W of RF power is used to excite the surface electrodes at the resonance frequency. For the wafer of thickness $L = 0.135$ mm, $\bar{S}_{yz} \approx 6.2 \times 10^{-4}$ to reach optimum modulation. This optimum strain level is reached if 11.9 mW $\times \left(\frac{6.2\times10^{-4}}{4.6\times10^{-5}}\right)^2 \approx 2.2$ W of RF power is used to excite the surface electrodes at the resonance frequency. For the wafer of thickness $L = 0.088$ mm, $\bar{S}_{yz} \approx 9.5 \times 10^{-4}$ to reach optimum modulation. This optimum strain level is reached if 12.6 mW $\times \left(\frac{9.5\times10^{-4}}{4.8\times10^{-5}}\right)^2 \approx 4.9$ W of RF power is used to excite the surface electrodes at the resonance frequency.

11 Ranging Performance of the Time-of-Flight Imaging System

In this section, we will express the ranging accuracy of the proposed imaging system as a function of the system parameters. Single target assumption per image sensor pixel will be made in the analysis (since most targets are optically opaque, resulting in a single reflection back to the receiver). The system parameters are: Optical transmitter power (P) illuminating the scene, frame rate of the image sensor (f_s), number of image sensor pixels (N), quantum efficiency of the pixels (QE), wavelength of the optical transmitter (λ_o), modulation frequency (f_m), depth of intensity modulation of the modulator (M), image sensor lens radius (L), target reflectivity (r), target distance (d_T). Let's assume homodyne detection is used, where the illumination intensity modulation frequency is equal to the modulator drive frequency. The phase of the intensity modulated light can be estimated by capturing four frames with different phases (through varying the transmitter phase). The electrons accumulated on an image sensor pixel per frame can be expressed as:

$$I_i = \mathrm{DC} + \mathrm{DC} \times \mathrm{M}\cos\Big(\Phi + \frac{(i-1)\pi}{2}\Big) + n_i;\ i \in \{1,2,3,4\} \tag{S52}$$

For the above expression, n_i are independent and identically distributed Gaussian random variables (capturing noise, assumed to be Gaussian to simplify analysis), where $n_i \sim \mathcal{N}(0, \mathrm{DC} + \sigma^2)$, σ captures the noise standard deviation of the sensor (i.e. sensor noise), and DC in the variance of the Gaussian random variable is due to shot-noise of the light source (also assumed to be Gaussian, which is true for higher levels of electrons accumulated per frame - Poisson convergence to Gaussian).

The phase carrying the distance information per pixel Φ can be estimated with $\tilde{\Phi}$ as follows:

$$\tilde{\Phi} = \tan^{-1}\Big(\frac{I_4 - I_2}{I_3 - I_1}\Big) \tag{S53}$$

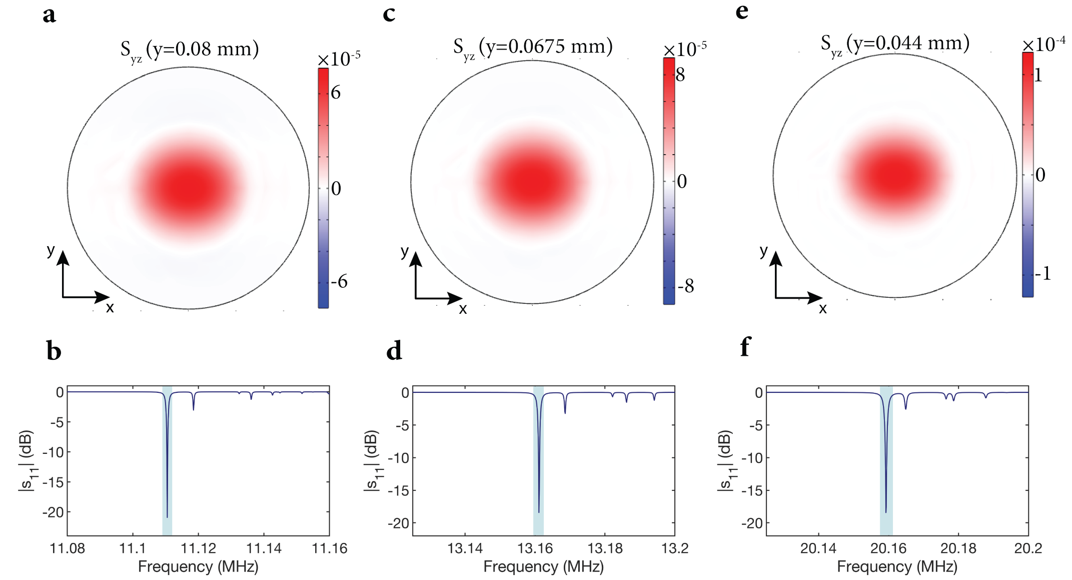

Fig. S11. Higher frequency of operation using thinner wafers. **a** LN Y89 wafer with a diameter of 25.4 mm and with thickness 0.16 mm is used. Top and bottom surface electrodes are centered on the wafer, having a diameter of 12.7 mm. S_{yz} strain profile for the plane that is parallel and 0.08 mm above the bottom wafer surface is shown when 2Vpp at a frequency of 11.1106 MHz is applied to the wafer surface electrodes. **b** Simulated $|s_{11}|$ of the wafer described in **(a)** with respect to 50 Ω, with the light blue region showing the excited mode. **c** LN Y89 wafer with a diameter of 25.4 mm and with thickness 0.135 mm is used. Top and bottom surface electrodes are centered on the wafer, having a diameter of 12.7 mm. S_{yz} strain profile for the plane that is parallel and 0.0675 mm above the bottom wafer surface is shown when 2Vpp at a frequency of 13.1611 MHz is applied to the wafer surface electrodes. **d** Simulated $|s_{11}|$ of the wafer described in **(c)** with respect to 50 Ω, with the light blue region showing the excited mode. **e** LN Y89 wafer with a diameter of 25.4 mm and with thickness 0.088 mm is used. Top and bottom surface electrodes are centered on the wafer, having a diameter of 12.7 mm. S_{yz} strain profile for the plane that is parallel and 0.044 mm above the bottom wafer surface is shown when 2Vpp at a frequency of 20.1592 MHz is applied to the wafer surface electrodes. **f** Simulated $|s_{11}|$ of the wafer described in **(e)** with respect to 50 Ω, with the light blue region showing the excited mode.

Without loss of generality, we assume $\Phi = 0$ to simplify the noise analysis. This assumption simplifies the expression to:

$$\tilde{\Phi} = \tan^{-1}\Big(\frac{I_4 - I_2}{I_3 - I_1}\Big) \approx \frac{n_4 - n_2}{2\text{DC} \times \text{M}} \tag{S54}$$

The distribution of the phase estimate $\tilde{\Phi}$ can now be expressed more generally as:

$$\tilde{\Phi} \sim \mathcal{N}\Big(\Phi, \frac{\text{DC} + \sigma^2}{2\text{DC}^2\text{M}^2}\Big) \tag{S55}$$

The distance estimate $\tilde{d}_T$ per pixel can be estimated as:

$$\tilde{d}_T = \frac{\tilde{\Phi} c}{4\pi f_m} \sim \mathcal{N}\left(d_T, \frac{\text{DC} + \sigma^2}{2\text{DC}^2\text{M}^2} \times \frac{c^2}{16\pi^2 f_m^2}\right) \tag{S56}$$

The shot-noise contribution to noise (DC) can be estimated as follows:

$$\text{DC} = \frac{\text{Pr}}{2\text{N}} \times \frac{\pi \text{L}^2}{2\pi d_T^2} \times \text{QE} \times \frac{1}{f_s \frac{hc}{\lambda_o}} \tag{S57}$$

For the expression above, c is the speed of light in vacuum, and h is Planck's constant. Lambertian reflectance is assumed for the target. The above analysis gives the ranging accuracy (modeled as a Gaussian distribution) as a function of the system parameters. Since four frames are used to estimate distance per pixel, the refresh rate of

the ToF estimate is $\frac{f_s}{4}$. The unambiguous imaging range is up to $\frac{c}{2f_m}$. Operation at a single frequency results in a trade-off between the ranging accuracy and the unambiguous imaging range (due to phase wrapping).

The trade-off between unambiguous imaging range and ranging accuracy can be broken through performing phase measurements at multiple frequencies. Assume M frequencies (f_k; $k \in \{1, 2, ..., M\}$) are used for the distance measurement, each resulting in a phase-shift of $\tilde{\Phi}_k$; $k \in \{1, 2, ..., M\}$ (estimated using equation (S53)), respectively. For an unambiguous imaging range of d_{max} (depends on M, the modulation frequencies, signal-to-noise ratio, and side-lobe levels), the most probable target location per pixel can be estimated as follows:

$$\tilde{d}_{TM} = \arg\max_{d} \mathfrak{Re}\left\{ \sum_{k=1}^{\mathrm{M}} e^{-j\frac{4\pi f_k \mathrm{d}}{c}} e^{j\tilde{\Phi}_k} \right\};\ d \in [0, d_{max}) \tag{S58}$$

For this analysis, shot-noise of the optical light source and sensor noise have been assumed to be the contributing noise sources.

11.1 Ranging accuracy of the time-of-flight imaging system used for the experiments

In this section, we will calculate the ranging accuracy of the ToF system used for the experiments, and compare against experimentally obtained results. If we know DC, σ, f_m, and M, we can calculate the standard deviation of the ranging accuracy using equation (S56). DC and M (as a percentage) are shown in Fig. S12 per pixel, and $f_m = 3.77$ MHz. The dark noise of the image sensor used is 13.8 electrons/second. For one of the targets (T_1), the expected standard deviation of the distance estimate per pixel can be computed for the region enclosed by the dashed white rounded rectangle shown in Fig. S12**c** as follows:

$$\Delta d_T = \sqrt{\frac{1}{\mathrm{U}} \sum_{u=1}^{\mathrm{U}} \frac{\frac{\mathrm{N_{frames}} \times \mathrm{DC}_u}{4} + \frac{\sigma^2 \times \mathrm{N_{frames}}}{f_s}}{2\left(\frac{\mathrm{N_{frames}} \times \mathrm{DC}_u}{4}\right)^2 \mathrm{M}_u^2} \times \frac{c^2}{16\pi^2 f_m^2}} \approx 1.06 \text{ m} \tag{S59}$$

For the expression above, $\mathrm{U} = 399,754$ is the number of pixels that exist in the region enclosed by the dashed white rounded rectangle in Fig. S12**c**, $\mathrm{N_{frames}} = 600$ is the total number of frames captured by the image sensor, $f_s = 10$ Hz is the frame rate of the image sensor, $f_m = 3.77$ MHz, M_u is the DoM per pixel in Fig. S12**c**, and DC_u is the average number of electrons per frame per pixel in Fig. S12**c**.

The histogram of the phase of the beat tone at 2 Hz for the enclosed region in Fig. S12**c** is shown in Fig. S12**d**. The standard deviation of the phase estimate per pixel is approximately 0.16 radians, which translates to a ranging standard deviation of $0.16 \times \frac{c}{4\pi f_m} \approx 1$ m. This experimentally obtained ranging accuracy per pixel agrees well with the estimated ranging accuracy of 1.06 m using the noise model.

We have verified in this section that the ranging accuracy model is consistent with experimentally obtained results. The ranging accuracy could be significantly improved by increasing the optical illumination power, increasing the depth of intensity modulation of the modulator, using higher frequency modulators, and increasing the photon collection aperture of the ToF system. The achievable ranging accuracy using the proposed system with optimized parameters is discussed in the next section.

11.2 Achievable ranging accuracy with single frequency operation

In this section, we will demonstrate the ranging accuracy of the proposed ToF imaging system for two different cases. In the first case, the modulation frequency $f_m = 3.77$ MHz, with the following parameters: P = 200 mW, $f_s = 60$ Hz (ToF frame rate of 15 Hz), $\mathrm{N} = 10^6$, QE = 70%, $\lambda_o = 905$ nm, M = 80%, L = 2 cm, r = 10%, $\sigma = 6$ (corresponding to a dark current of 360 electrons/second). In the second case, the modulation frequency $f_m = 20$ MHz, with the other parameters remaining the same. It is assumed that the modulator is placed in front of the image sensor pixels, so that the light capturing aperture is limited by the lens radius L. It is further assumed that the optical source illuminating the scene field-of-view matches the image sensor field-of-view. If the divergence angle of the illuminator is taken to be 30 degrees, the optical intensity at a distance of 50 cm from the optical source is approximately 0.08 $\mathrm{mWcm^{-2}}$. This exposure level is below the maximum permissible exposure (MPE) limit when eye-safety is taken into account for continuous mode operation[15].

Assuming a target is placed between 50 cm and 7.5 m (unambiguous imaging range for single frequency operation at 20 MHz) from the imaging system, the target localization standard deviation as a function of the target distance per pixel is shown in Fig. S13 for the two different modulation frequencies using equations (S56) and (S57).

11.3 Achievable ranging accuracy with multi-frequency operation

Assume three different image sensors, each integrated with a modulator operating at a different frequency, and each operating in the homodyne mode as described in the previous section. Each image sensor has its own optical

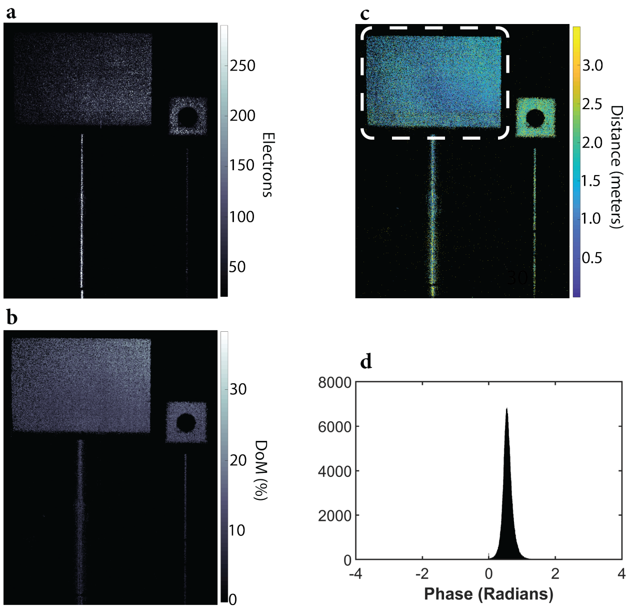

Fig. S12. Ranging accuracy and statistics for the time-of-flight imaging experiment. **a** Accumulated average number of electrons per pixel per frame of the image sensor when normalized with the camera gain of 23 dB. **b** The DoM at the beat tone of 2 Hz per pixel is shown. **c** Reconstructed depth map seen by the camera. Reconstruction is performed by mapping the phase of the beat tone at 2 Hz to distance. **d** Histogram of the phase of the beat tone at 2 Hz for the pixels in the region enclosed by the dashed white rounded rectangle in **(c)**. 399,754 pixels exist in this region.

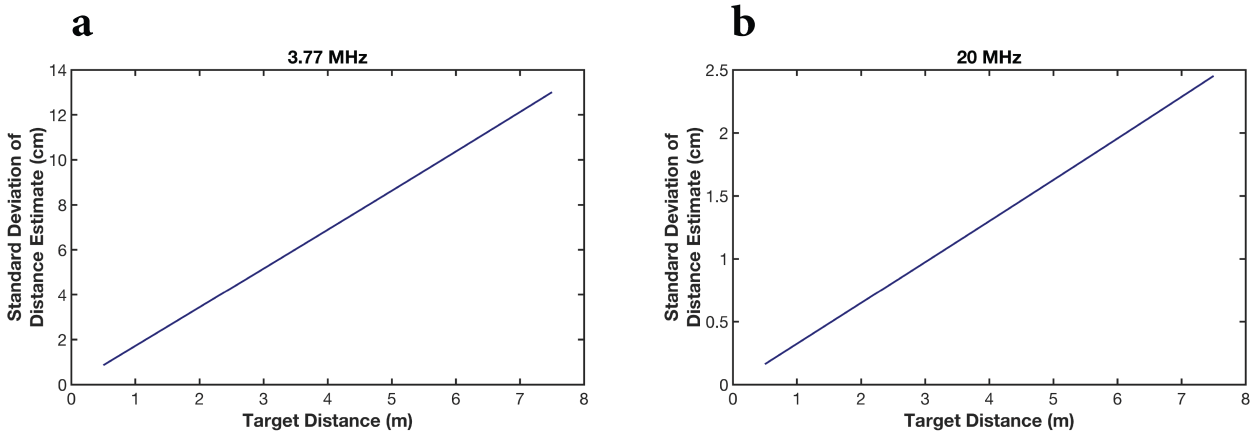

Fig. S13. Ranging accuracy of the time-of-flight imaging system using single frequency operation as a function of target location. It is assumed that the illuminated target in the scene is opaque and has Lambertian reflectance. **a** 3.77 MHz modulation frequency case. **b** 20 MHz modulation frequency case.

illuminator, with slightly different optical wavelengths (to prevent interference for the image sensors) and optical intensity modulation frequencies. Each illuminator has an optical illumination power of 67 mW. If the image sensors are in close proximity to each other compared to the target distance, parallax will be negligible, allowing the ToF data generated from each image sensor to be easily fused.

Each image sensor has a spectral filter, so that DC component is only affected by its own source, and not from other light sources to prevent excess noise from other illuminators. Advantage is taken of multi-frequency operation to rely on high frequencies (20 MHz), while having an unambiguous imaging range required for many applications (e.g. robotics, drones). The plots in Fig. S14 show the attainable performance for this ToF system. The parameters used are as follows: P = 67 mW, f_s = 60 Hz (ToF frame rate of 15 Hz), N = 10^6, QE = 70%, λ_o = 905 nm, M = 80%, L = 2 cm, r = 10%, σ = 6 (corresponding to a dark current of 360 electrons/second) for each of the modulation frequencies of 11 MHz, 13 MHz, and 20 MHz. Assuming a target is placed between 50 cm and 80 m (d_{max} = 80 m) from the imaging system, the ranging standard deviation as a function of the target distance per pixel is shown in Fig. S14. For estimating the ranging accuracy for this plot, Monte Carlo simulations were performed 1,000 times and averaged. For each simulation, the target distance was estimated using equation (S58).

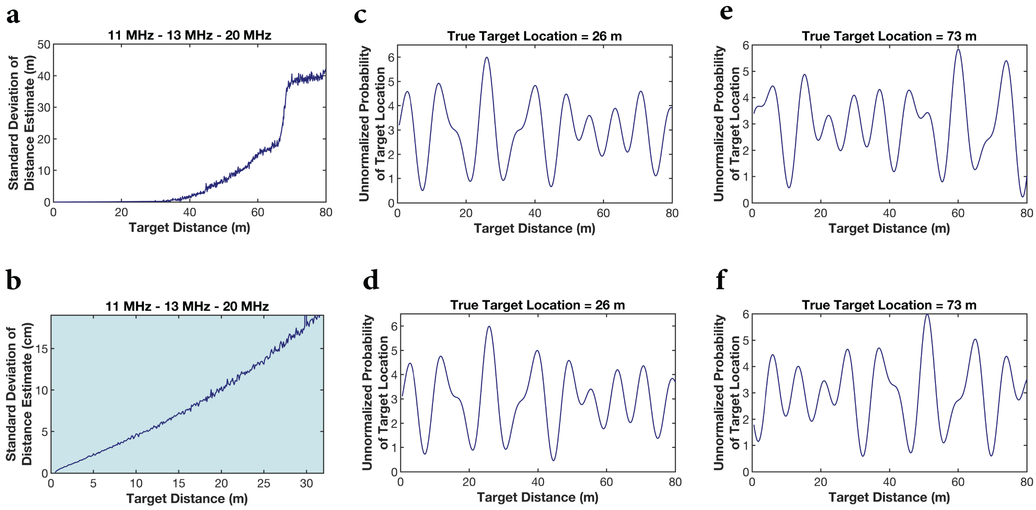

Fig. S14. Ranging accuracy of multi-frequency operation with intensity modulation frequencies of 11 MHz, 13 MHz, and 20 MHz. a Ranging accuracy of multi-frequency operation with three image sensors. **b** Zoomed in version of the ranging accuracy of high performance region (up to 30 m) in **(a)**. **c** Simulated target location likelihood using equation (S58), assuming a target is placed 26 m away from the imaging system. Reconstruction resolution is 1 cm. Estimated target location of 26.03 m agrees well with the actual target distance of 26 m. **d** Another simulated target location likelihood using equation (S58), assuming a target is placed 26 m away from the imaging system. Reconstruction resolution is 1 cm. Estimated target location of 25.81 m agrees well with the actual target distance of 26 m. **e** Simulated target location likelihood using equation (S58), assuming a target is placed 73 m away from the imaging system. Reconstruction resolution is 1 cm. Estimated target location of 60.06 m is significantly different than the actual target distance of 73 m. **f** Another simulated target location likelihood across distance using equation (S58), assuming a target is placed 73 m away from the imaging system. Reconstruction resolution is 1 cm. Estimated target location of 50.96 m is significantly different than the actual target distance of 73 m.

Fig. S14**a** shows good performance for the ranging accuracy up to approximately 30 m, after which the performance degrades significantly. This is to be expected for this estimation, since maximum likelihood estimation is used, which is non-linear. Non-linear estimators have this threshold effect, where performance drops substantially below some signal-to-noise ratio[16]. The simulations, however, show that approximately 10 cm ranging accuracy can be reached up to 30 m with the proposed system and parameters.

References

1. Yariv, A. & Yeh, P. *Optical Waves in Crystals* (Wiley New York, (1984)).
2. Andrushchak, A. *et al.* Complete sets of elastic constants and photoelastic coefficients of pure and MgO-doped lithium niobate crystals at room temperature. *Journal of Applied Physics* **106** ((2009)).
3. Dixon, R. Acoustic diffraction of light in anisotropic media. *IEEE Journal of Quantum Electronics* **3,** 85–93 ((1967)).
4. Harris, S. & Wallace, R. Acousto-optic tunable filter. *JOSA* **59,** 744–747 (1969).
5. *Thorlabs website (EO-AM-R-20-C1). Available online:* https://www.thorlabs.com. Accessed: 21 March 2021.
6. *Thorlabs website (EO-PC-550). Available online:* https://www.thorlabs.com. Accessed: 21 March 2021.
7. Mohseni, H. *Electroabsorption modulator for depth imaging and other applications* US Patent 10,254,407. Apr. 2019.
8. Huang, Y.-W. *et al.* Gate-tunable conducting oxide metasurfaces. *Nano Letters* **16,** 5319–5325 ((2016)).
9. Park, J. *et al.* All-solid-state spatial light modulator with independent phase and amplitude control for three-dimensional LiDAR applications. *Nature Nanotechnology* **16,** 69–76 ((2021)).
10. Pinnow, D. Guide lines for the selection of acoustooptic materials. *IEEE Journal of Quantum Electronics* **6,** 223–238 (1970).
11. Mytsyk, B., Demyanyshyn, N., Andrushchak, A. & Buryy, O. Photoelastic Properties of Trigonal Crystals. *Crystals* **11,** 1095 (2021).

12. Yang, J., Long, J. & Yang, L. First-principles investigations of the physical properties of lithium niobate and lithium tantalate. *Physica B: Condensed Matter* **425,** 12–16 (2013).

13. Gachon, D. *et al. P1H-1 LiNbO3-LiNbO3 high overtone bulk acoustic resonator exhibiting high Qf product* in *2007 IEEE Ultrasonics Symposium Proceedings* (2007), 1417–1420.

14. Pijolat, M. *et al. Large Qxf product for HBAR using Smart Cut™ transfer of LiNbO 3 thin layers onto LiNbO 3 substrate* in *2008 IEEE Ultrasonics Symposium* (2008), 201–204.

15. Maini, A. K. *Lasers and Optoelectronics: Fundamentals, Devices and Applications* (John Wiley & Sons, 2013).

16. Bell, K. L., Steinberg, Y., Ephraim, Y. & Van Trees, H. L. Extended Ziv-Zakai lower bound for vector parameter estimation. *IEEE Transactions on Information Theory* **43,** 624–637 (1997).
